# Comprehensive Analysis of Heat and Water Exchanges in the Human Lungs

**DOI:** 10.3389/fphys.2021.649497

**Published:** 2021-06-08

**Authors:** Benoit Haut, Antoine Nonclercq, Alexandra Buess, Jérémy Rabineau, Clément Rigaut, Benjamin Sobac

**Affiliations:** ^1^Ecole Polytechnique de Bruxelles, Transfers, Interfaces and Processes (TIPs), Université libre de Bruxelles, Brussels, Belgium; ^2^Ecole Polytechnique de Bruxelles, Bio, Electro and Mechanical Systems (BEAMS), Université libre de Bruxelles, Brussels, Belgium; ^3^Université de Pau et des Pays de l'Adour, E2S UPPA, CNRS, Total, LFCR, Anglet, France

**Keywords:** asthma, cystic fibrosis, evaporation, exercising, heat and mass transfers, scaling, lung, model

## Abstract

This work presents a new mathematical model of the heat and water exchanges in the human lungs (newborn to adult). This model is based on a local description of the water and energy transports in both the lumen and the surrounding tissues, and is presented in a comprehensive, dimensionless framework with explicitly stated assumptions and a strong physiological background. The model is first used to analyze and quantify the key phenomena and dimensionless numbers governing these heat and water exchanges and then it is applied to an adult in various situations (varying atmospheric conditions, exercising…). The results highlight several interesting physiological elements. They show that the bronchial region of the lungs is able to condition the air in all the considered situations even if, sometimes, for instance when exercising, distal generations have to be involved. The model also shows that these distal generations are super-conditioners. Moreover, the results quantify the key role of the submucosal glands in mucus hydration. They also show that, during expiration, a significant cooling of the air and condensation of water occur along the respiratory tract as the vascularization of the tissues surrounding the airways is not able to maintain these tissues at body temperature during inspiration. Due to the interaction between several phenomena, it appears that the ratio of the amount of water returned to the mucosa during expiration to the amount extracted during inspiration is almost independent of the breathing conditions (around 33%). The results also show that, in acute situations, such as suffering from a pathology with airway dysfunction, when being intubated or when exercising above an intensity threshold, the heat and water exchanges in the lungs may be critical regarding mucus hydration. In proximal generations, the evaporation may overwhelm the ability of the submucosal glands to replenish the airway surface liquid with water. In some situations, the cooling of the mucosa may be very important; it can even become colder than the inspired air, due to evaporative cooling. Finally, the results show that breathing cold air can significantly increase the exchanges between the lungs and the environment, which can be critical regarding disease transmission.

## 1. Introduction

**Figure 1:**
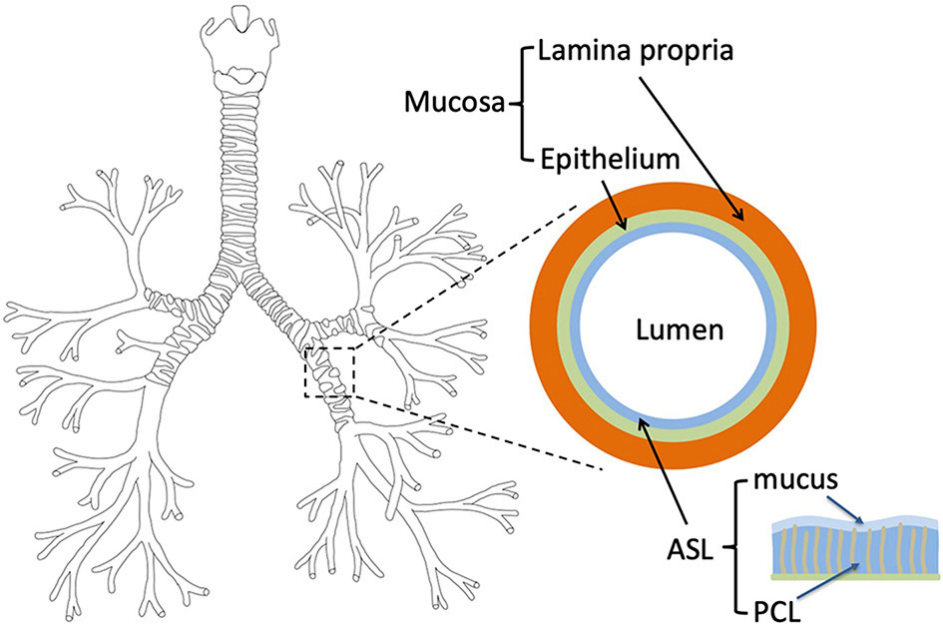
**Left**: tree structure of the bronchial region of the human lungs. **Right**: layered representation of the bronchial mucosa, with the ASL, the epithelium and the lamina propria.

In humans and other mammals, the respiratory system is divided into an upper and a lower respiratory tract. The upper tract includes the nose, the nasal cavities, the pharynx, and the larynx, while the lower tract is composed of the lungs. The lungs form a dichotomous branching tree in which each level of subdivision is called a generation (see Figure 1); the trachea being the first generation. The lungs are themselves divided in two regions: the bronchial region, composed of airways (trachea, bronchi, bronchioles…) and designed for air transport, and the alveolar region, composed of acini (i.e., clusters of alveoli) and designed for the exchange of the respiratory gases. In a human adult, the bronchial region is approximately made up of 17 generations (Weibel, 1963; Weibel et al., 2005). As sketched in Figure 1, the mucosa of the airways is composed of a single layer of epithelial cells, the epithelium, overlying a layer of a loose connective tissue, the lamina propria (Jeffery et al., 1998). Moreover, in order to protect the lungs from particles and pathogens, the epithelium is covered with a thin liquid layer, the Airway Surface Liquid (ASL). The ASL is composed of two sublayers: the mucus layer and the PeriCiliary Layer (PCL) (King, 2006; Button et al., 2012). The mucus layer is a sol-like substance made of water and large macromolecules (mucins), in contact with the air and where foreign inhaled particles and pathogens are trapped in. The mucus layer is overlying the PCL, also a sol-like substance, between the mucus layer and the epithelium. Furthermore, some cells composing the bronchial epithelium are ciliated and, by beating in the PCL, these cilia displace the mucus from the distal part of the bronchial region to the top of the trachea, where it is swallowed or expectorated (Chateau et al., 2019). This mucociliary clearance constitutes a fundamental defense mechanism of the body against pathogens. A similar mucosa is also present in the upper tract.

Besides the exchange of O_2_ and CO_2_ with the environment, another important function of the respiratory tract is to condition the air before it reaches the alveolar region (i.e., to warm it to the body temperature and to saturate it with water) (Walker and Wells, 1961). This conditioning occurs because the inspired air is usually colder and dryer than the mucosa with which it is in contact in the respiratory tract. Consequently, energy is transferred from the tissues surrounding the respiratory tract to the ASL–lumen interface, in order to heat the air in the lumen and to evaporate water contained in the ASL. This process leads globally to an extraction of heat and water from the body by the respiration. The maximal amounts of heat and water that could be extracted per unit of time from the body by the respiration can be calculated easily as they correspond to a hypothetical situation in which the expired air would be saturated with water and at the body temperature. For instance, if we consider a human adult, inspiring and expiring 15 times per minute a volume of 500 ml of ambient air at a temperature of 20°C and a relative humidity of 0.6, we can easily calculate that the maximal amount of heat and the maximal amount of water that could be extracted, per unit of time, from the body by the respiration are approximately 13 W and 360 ml day^−1^, respectively (see the Supplementary Material for more details). These values are non-negligible when compared to the basal metabolic rate (approximately 80 W) and the total hydric loss per day (approximately 2 l) of a human adult (Roza and Shizgal, 1984; Dmitrieva and Burg, 2011). Consequently and as pointed out by several studies (Havenith, 2005; Dmitrieva and Burg, 2011; Sobac et al., 2020), the respiratory tract is not insignificant regarding heat and water losses. Moreover, it can be easily evaluated that the power used to evaporate water accounts for approximately 80% of the total power extracted from the body by the ventilation (except when breathing very cold air). As a consequence, the amount of energy and the amount of water extracted from the body by the ventilation are almost proportional to each other (see the Supplementary Material).

The conditioning of the air by the respiratory tract is physiologically very important because it protects the alveolar membrane from thermal injury and, by keeping this membrane wet, it permits the carbon dioxide excretion to occur rapidly (Walker and Wells, 1961). Moreover, the way the air conditioning is realized in the respiratory tract closely relates to a proper operation of the mucociliary clearance. Several works have shown that, in human breathing by the nose, this air conditioning takes place very largely in the upper respiratory tract and in the proximal generations of the lungs (i.e., in the trachea and in the few generations downstream), except in acute situations (Walker and Wells, 1961; Mcfadden et al., 1985). Many authors pointed out the importance of this feature (Estes and Meduri, 1995; Williams et al., 1996). They mention that, if the upper respiratory tract and the proximal generations of the lungs were not able to condition the air fully, the heat and water exchanges that would take place in the distal generations of the lungs could lead to a reduced efficiency of the mucociliary clearance in these generations, due to the decreased mobility of the mucus induced by its dehydration. This is less likely to occur in the proximal generations, given the abundant presence of submucosal glands, secreting mucus, in these generations (Widdicombe, 2002; Joo et al., 2006). It is important to insist on the fact that, even when breathing at rest and by the nose, a non-negligible part of the air conditioning is realized in the proximal generations of the lungs (i.e., the air is not fully conditioned by the upper respiratory tract). It is for instance clearly shown by the experimental data of Mcfadden et al. (1985): these authors have measured that, when a human adult at rest breathes in a room with an air at a temperature of 27°C and a relative humidity of 40%, the temperature of the air at the top of the trachea during inspiration is approximately 33°C and the air is not yet at the body temperature in the sixth generation. Moreover, they have shown that a significant cooling of the air is happening in the lungs during expiration. They explained this by hypothesizing that the temperature of the mucosa is markedly decreased by the heat extraction during inspiration (Mcfadden, 1992). Consequently, during expiration, the air saturated with water and at the body temperature flowing out of the alveolar region is in contact with a significantly colder mucosa, leading to this non-negligible cooling. It is worth to mention that this cooling during expiration is also shown experimentally by Dery (1973), mentioned by Walker and Wells (1961) and calculated in Karamaoun et al. (2018), Sobac et al. (2020), and Wu et al. (2014). Of course, when breathing by the mouth such as when exercising, a large part of the upper respiratory tract is bypassed and the role of the lungs in conditioning the air is increased.

With the different above-mentioned elements, we understand that a drawback of the air conditioning by the respiratory tract is that, in some acute situations, it can induce problems in the lungs due to heat and/or hydric losses. For instance, it is well-known that inspiring cold and dry air when exercising can lead to a dehydration of the ASL, inducing cell shrinkage, release of inflammatory mediators, airway smooth muscle constriction and both physical and chemical activation of cough receptors (Banner et al., 1984; Kippelen and Anderson, 2013). Similar issues have been pointed out in several situations: when an invasive respiration is realized (intubation during an operation or tracheotomy) (Dery, 1973; Jackson, 1996; Rathgeber et al., 1996; Ryan et al., 2002), when taking care of a premature child (Jarreau, 2016), when ASL replenishment with water is impaired in cystic fibrosis (Karamaoun et al., 2018), when an asthmatic person is exercising or simply breathing in cold and dry air (Gilbert and Mcfadden, 1992; Mcfadden, 1992; Koskela, 2007; Cote et al., 2018; D'Amato et al., 2018).

Despite their clinical interest, the analysis of the heat and water exchanges in the lungs has been the subject of a limited number of modeling works (Saidel et al., 1983; Ferron et al., 1985; Ingenito et al., 1986; Scherer and Hanna, 1986; Tsu et al., 1988; Daviskas et al., 1990; Tsai et al., 1990; Tawhai and Hunter, 2004; Warren et al., 2010; Wu et al., 2014; Karamaoun et al., 2018; Sobac et al., 2020). It is probably due to the complex nature of the problem and to the scarcity of the clinical data to confront the models with. In these works, the transport of heat in the tissues surrounding the bronchi, which has a crucial importance on the dynamics of the system, is often represented in a simplified manner, either by considering an isothermal mucosa–lumen interface at the body temperature, or by imposing a temperature profile at this interface in order to reproduce qualitatively experimental results. In addition, even if it is known that the enhanced temperature and concentration gradients in the developing part of the flow in an airway can have a huge impact on the overall transfers in this airway (Pedley et al., 1970b), the flow of the air in the lungs is also often represented in a simplified manner in these works, not taking precisely into account its non-establishment in the large airways. Several works have nevertheless used Computational Fluid Dynamics (CFD) simulations to fully analyze the flow and the transfers in the lungs (see for instance Wu et al., 2014), but on specific geometries obtained by CT-Scan, preventing a large generality of the results. More generally, the analysis of the works cited above shows that there is a real need for a complete and detailed analysis of the exchanges of water and heat in the human lungs, in order to understand what the key governing phenomena are and how they depend on the breathing conditions.

The general objective of this work is to give further insights into the complexity of heat and water exchanges in the human lungs (from a newborn to an adult). Our first goal is to provide a new mathematical model, easy to use and to implement, allowing a comprehensive analysis of these exchanges, based on their local description in the lumen and in the surrounding tissues, with a strong physiological background and in a general framework. Our second goal is then to use this model to give more insights into the complexity of the heat and water exchanges in the lungs. Notably, we want to highlight the key phenomena and the key dimensionless numbers governing these exchanges, to analyze how these exchanges are distributed in the lungs and how these dimensionless numbers are related. Finally, our third goal is to use the model to provide new elements to understand the heat and water exchanges in the lungs of a human adult breathing in various situations, such as when being intubated, or suffering from cystic fibrosis, or exercising possibly in cold air, or being in altered pressure conditions (diving, climbing to the top of a high mountain).

## 2. Mathematical Model

In the model, any temperature is referred to by the symbol *T* (K) and any concentration of water in air by the symbol *C* (molm^−3^). The physicochemical properties involved in the model are considered constant (i.e., independent of the local value of the temperature or the water concentration in the air). They are calculated using equations given in Sobac et al. (2015) and considering that the thermal properties of the ASL, the epithelium and the tissues surrounding the airways are those of liquid water (Warren et al., 2010). They are calculated at the body temperature, written *T*_*b*_ and taken equal to 37°C, and, for the physicochemical properties of the air, at a relative humidity of 100% and a pressure of 101,325 Pa (except in two cases, detailed below). We verified that, for all the cases considered in this article, no significant difference would have been observed on the results if these properties had been evaluated using the temperature and the relative humidity of the air at the top of the trachea during inspiration. The saturation concentration of water in air at the temperature *T*, *C*_sat_(*T*), is calculated using the Clausius-Clapeyron equation, with a reference temperature equal to *T*_*b*_, considering that the air is an ideal gas. As the molar fraction of mucins in the mucus is much smaller than 1 (Lai et al., 2009), we assume, as in other works, that the saturation pressure of the mucus layer is the one of pure water (Wu et al., 2014). The values of the different physicochemical properties used in the model are provided in the Supplementary Material.

The temperature of the air and the water vapor concentration at the top of the trachea, during inspiration, are written *T*_0_ and *C*_0_, respectively. The relative humidity at the top of the trachea during inspiration is RH_0_ = *C*_0_/*C*_sat_(*T*_0_). Dimensionless temperature and concentration are defined as:

(1) $$\begin{array}{l} \tilde{T}=\frac{T-T_{0}}{T_{b}-T_{0}}\text{ and }\tilde{C}=\frac{C-C_{0}}{C_{\text{sat}}\left(T_{b}\right)-C_{0}} \end{array}$$

The expiration duration, *t*^exp^ (s), is equal to the inspiration duration, *t*^insp^ (s), times a factor γ: *t*^exp^ = γ*t*^insp^. At rest, the typical value of γ is 2. The total duration of a respiratory cycle is *t*^tot^ = *t*^insp^ + *t*^exp^ = (1 + γ)*t*^insp^. The inspiration and expiration flow rates are written *Q*^insp^ and *Q*^exp^ (m^3^ s^−1^), respectively. Both are defined as being positive.

Concerning the geometry, we use the common so-called “Weibel A” representation (Weibel, 1963), which considers the bronchial region of the lungs as a dichotomous tree. The generations are numbered, starting from the trachea (number 1), and the total number of generations in the bronchial tree is written *n*. The number of airways belonging to generation *i* is 2^*i*−1^. All the airways are right circular cylinders and all the airways belonging to the same generation have the same dimensions. Therefore, each airway in generation *i* is characterized by one length, *L*_*i*_ (m), and one inner radius, *R*_*i*_ (m). It divides in two identical airways belonging to generation *i* + 1. This division is called a bifurcation. As detailed below, two different approaches are used to express the values of *n*, *L*_*i*_, and *R*_*i*_ (see section 2.2.2 and **Table 2**).

Finally, we assume that the dimensions of the airways are constant during a respiratory cycle. This assumption is supported by the fact that the airways contributing mainly to heat and water exchanges are the proximal ones and that the dimensions of these airways experience little variations during a respiratory cycle, due to the significant presence of cartilage around them (Haverkamp et al., 2005).

In what follows, our model is presented in two stages. First, the transfers at the scale of a single airway are modeled (in the lumen and in the surrounding tissues). Then, on this basis, a global model of the heat and water exchanges, at the scale of the lungs, is developed.

### 2.1. Heat and Water Exchanges in a Single Airway

**Figure 2:**
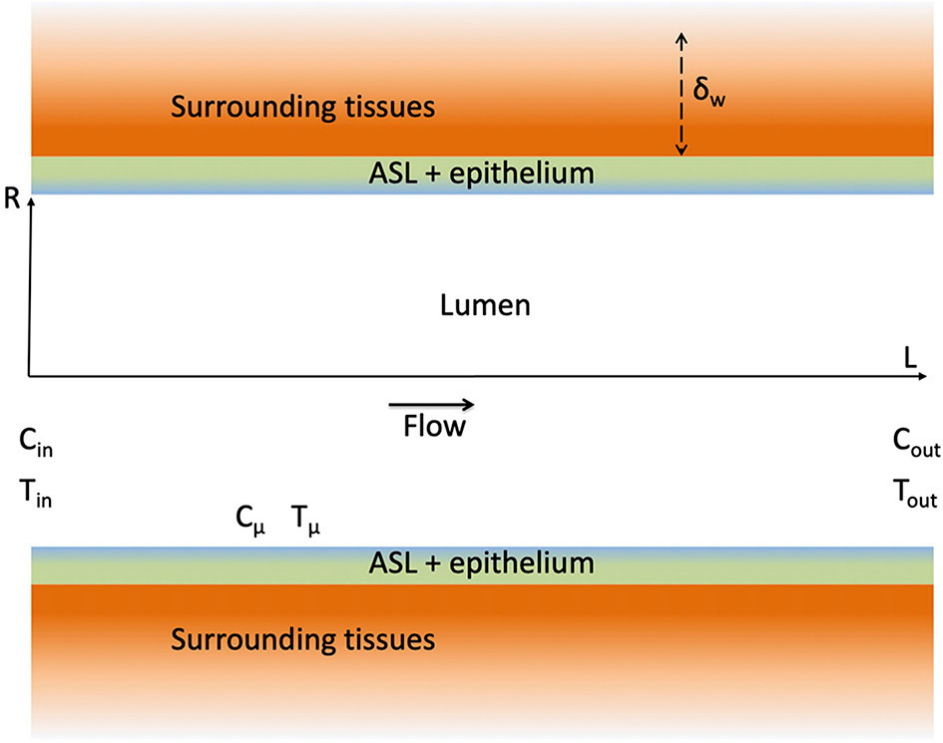
Schematic representation of an airway and the surrounding tissues. The figure is not on scale.

In this section, a single airway of length *L* and radius *R* is considered (see Figure 2). Our modeling approach is fully described in the Supplementary Material and only its key elements are presented here for the sake of conciseness.

#### 2.1.1. Transfers in the Lumen

Our approach is based on a local description of the transfers of momentum, heat and water vapor in the lumen of the airway. As detailed in the Supplementary Material, several assumptions are made, especially (i) that the air in the lumen is Newtonian and incompressible, (ii) that the transports in the lumen are axisymmetric and in a steady state (which of course differs depending on whether inspiration or expiration is considered) and (iii) that the water vapor concentration and the temperature at the ASL–lumen interface, written respectively *C*_μ_ and *T*_μ_ (see Figure 2), are independent of the axial position in the airway. The latter assumption is motivated by the fact that, in all the results presented in this paper, the dimensionless water concentration and temperature in the lumen exhibit small variations (less than 10%) in a single airway, during inspiration and expiration. These different assumptions make it possible to write 2D equations of continuity, momentum, heat and water vapor transports in the lumen, and their boundary conditions (Supplementary Equations 3–13 in the Supplementary Material). It is worth to mention that, in the proximal generations of the lungs, the flow-establishment length is usually significantly larger than the length of the airways, and that this undeveloped character of the flow in these proximal airways has a strong impact on the transfers (Pedley et al., 1970a,b). As detailed in the Supplementary Material, this is taken into account in our approach, as we impose a constant axial velocity at the inlet of the airway (Supplementary Equation 9 in the Supplementary Material) and as we solve the continuity and momentum transport equations in the airway. As steady states are assumed during inspiration and expiration, *Q*^exp^ and *Q*^insp^ are constant. Consequently, as the volume of inspired air is equal to the volume of expired air, *Q*^exp^ = *Q*^insp^/γ.

First, as described in the Supplementary Material, averaging the local mass transport equation for the water vapor in the lumen of the airway (Supplementary Equation 7 in the Supplementary Material) allows writing:

(2) $$\begin{array}{l} {\tilde{C}}_{\text{out}}=\frac{{\tilde{C}}_{\text{in}}-{\tilde{C}}_{\mu }}{\text{Ψ}}+{\tilde{C}}_{\mu } \end{array}$$

with

(3) $$\begin{array}{l} \text{Ψ}=\exp\left(4\beta \frac{\text{Sh}}{\text{Re Sc}}\right) \end{array}$$

${\tilde{C}}_{\text{in}}$ and ${\tilde{C}}_{\text{out}}$ are the averages of the dimensionless water concentration on the flow cross-section at the inlet and at the outlet of the airway, respectively (see Figure 2), while ${\tilde{C}}_{\mu }$ is the dimensionless version of *C*_μ_. Ψ is a dimensionless number characterizing the ability of the transport phenomena within the lumen of the airway to condition the air. If Ψ → ∞, ${\tilde{C}}_{\text{out}}\to {\tilde{C}}_{\mu }$, i.e., the conditioning occurs to the maximal possible extent, and, if Ψ → 1, ${\tilde{C}}_{\text{out}}\to {\tilde{C}}_{\text{in}}$, i.e., no water transfer occurs. Ψ is expressed by Equation (3), involving four dimensionless numbers: Sh, Re, Sc, and β. Sh, the Sherwood number, is a dimensionless mass transfer coefficient between the ASL and the lumen. It is defined as Sh = *kR*/*D*, with *k* (m s^−1^) the corresponding dimensional mass transfer coefficient (defined in Supplementary Equation 18 in the Supplementary Material and assumed independent of the axial position in the airway) and *D* (m^2^ s^−1^) the diffusion coefficient of water in air. Re = 2*Rv*_av_/ν is the Reynolds number of the flow in the airway, with ν (m^2^ s^−1^) the kinematic viscosity of the air and *v*_av_ (m s^−1^) the flow rate of air in the airway divided by the area of its cross-section, π*R*^2^. Finally, Sc = ν/*D* is the Schmidt number of the air, equal to 0.63 at body temperature and at a relative humidity of 100%, and β = *L*/*R* is the aspect ratio of the airway. Note that the physical meaning and the range of values of all the dimensionless numbers involved in our model are summarized in Table 1.

**Table 1:** Summary of the dimensionless numbers appearing in the model.

**Definition**	**Range of values**	**Physical meaning**
γ = *t*^*exp*^/*t*^*insp*^	1–2	Ratio of the expiration duration to the inspiration duration
β = *L*/*R*	3–16	Aspect ratio of an airway
Sc = ν/*D*	0.63	Schmidt number of the air, defined as the ratio of momentum diffusivity to mass diffusivity
Pr = ν/α	0.72	Prandtl number of the air, defined as the ratio of momentum diffusivity to thermal diffusivity
Re = 2*Rv*_av_/ν	1–10^4^	Reynolds number of the flow in an airway, defined as the ratio of inertial forces to viscous forces
Sh = *kR*/*D*	1–20	Dimensionless mass transfer coefficient in an airway (Sherwood number)
		For Re/β > 1, it can be related to Sc and Re using Equation (4).
Nu = *uR*/λ	1–20	Dimensionless heat transfer coefficient in an airway (Nusselt number)
$\text{Ψ}=\exp\left(4\beta \frac{\text{Sh}}{\text{Re Sc}}\right)$	1–10^5^	Ψ measures the ability of the mass transport phenomena within the lumen of an airway to condition the air.
		Ψ ≫ 1 means a high ability of an airway to condition the air.
		In the simplified framework, ${\text{Ψ}}_{i}^{\text{insp}}$ can be expressed as a sole function of ${\text{Re}}_{1}^{insp}/\beta$ and *i* using Equation (21).
$\bar{\text{Ψ}}=\exp\left(4\beta \frac{\text{Nu}}{\text{Re Pr}}\right)$	1–10^5^	$\bar{\text{Ψ}}$ characterizes the ability of the heat transport phenomena within the lumen of an airway to condition the air.
$\Lambda =\frac{R\lambda_{w}\left(T_{b}-T_{0}\right)}{L_{m}{\text{Sh}}^{\text{insp}}D\left(C_{\text{sat}}\left(T_{b}\right)-C_{0}\right)\sqrt{\alpha_{w}t_{w}}}$	0.03–0.3	Λ compares the ability of the vascularization to reheat the tissues composing the wall of an airway and the intensity of the heat withdrawal to evaporate water in the ASL.
		Λ ≫ 1 means a high ability of the vascularization to maintain the tissues at body temperature.
$\Phi =\frac{{\text{Nu}}^{\text{insp}}}{{\text{Sh}}^{\text{insp}}}\frac{\lambda }{DL_{m}}\frac{T_{b}-T_{0}}{C_{\text{sat}}\left(T_{b}\right)-C_{0}}$	0.10–0.15	Φ characterizes the relative importance of the two mechanisms in the air conditioning: the heating of the air up to body temperature and the saturation of the air with water.
Λ′	0.05–0.5	Dimensionless number with the same physical meaning than Λ.
		It appears in the simplified framework and it is given by Equation (23).
Θ	0.46	Limit of Λ′ for Re/β → ∞. It is given by Equation (24).
ϕ/ψ	0.3–1	Ratio of the increase of the cardiac output to the increase of the ventilation rate during an effort

*Typical range of values at atmospheric pressure. The physicochemical parameters are evaluated at 37°C and (for those related to the air) at a relative humidity of 100%*.

We see in Equation (3) that Ψ increases if βSh/Re ∝ β*k*/*v*_av_ increases. This is logical: the more the airway is slender (i.e., the larger β) and the more the velocity of the air is small compared to *k*, a “transfer velocity” of water between the wall of the airway and the lumen, the more the transport phenomena within the airway are efficient in terms of air conditioning.

**Figure 3:**
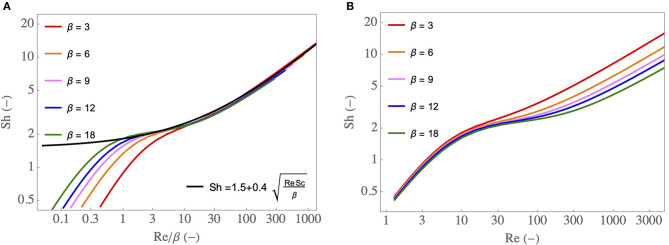
**(A)** Sh as a function of Re/β, for various β, as well as an approximate expression Sh = $1.5+0.4\sqrt{\text{Re Sc}/\beta }$. **(B)** Sh as a function of Re, for various β.

A dimensional analysis of the local transport equations in the lumen of the airway and their boundary conditions show that, for a fixed value of Sc, Sh is a sole function of Re and β. Therefore, as described in the Supplementary Material, CFD simulations of these equations can be used to construct a correlation relating Sh to Re and β: Sh = *f*(Re, β). In Figure 3A, results of these simulations are presented: Sh is plotted as a function of Re/β, for different values of β. The ranges of Re and β values considered cover the different situations encountered in section 3. Several interesting features can be observed on this figure. First, the results show that, for Re/β → ∞, Sh becomes proportional to $\sqrt{\text{Re}/\beta }$. This 1/2 exponent is typical from a “boundary layer” regime of mass transfer at a surface with a “plug flow” over it (Clift et al., 1978; Schlichting and Gersten, 2000). In our situation, by boundary layer regime we mean that the concentration gradients are located near the ASL–lumen interface in the entire airway. The results also show that, for Re/β → 0, Sh becomes proportional to Re (and independent of β). It is evidenced clearly in Figure 3B. This is typical of a “diffusion limited” regime of mass transfer with, in our situation, the concentration gradients spreading immediately over the entire flow cross-section of the airway. Between Re/β ≃ 1 and Re/β ≃ 10, Sh is almost constant (value close to 1.5–2). This transition between a diffusion limited regime and a boundary layer regime of mass transfer corresponds to a physical situation in which the boundary layers developing on opposite sides of the airway meet eventually at the center of the latter. From a practical point of view and as shown in Figure 3A, when Re/β > 1, Sh can be well-approximated by:

(4) $$\begin{array}{l} \text{Sh}=1.5+0.4\sqrt{\frac{\text{Re Sc}}{\beta }} \end{array}$$

**Figure 4:**
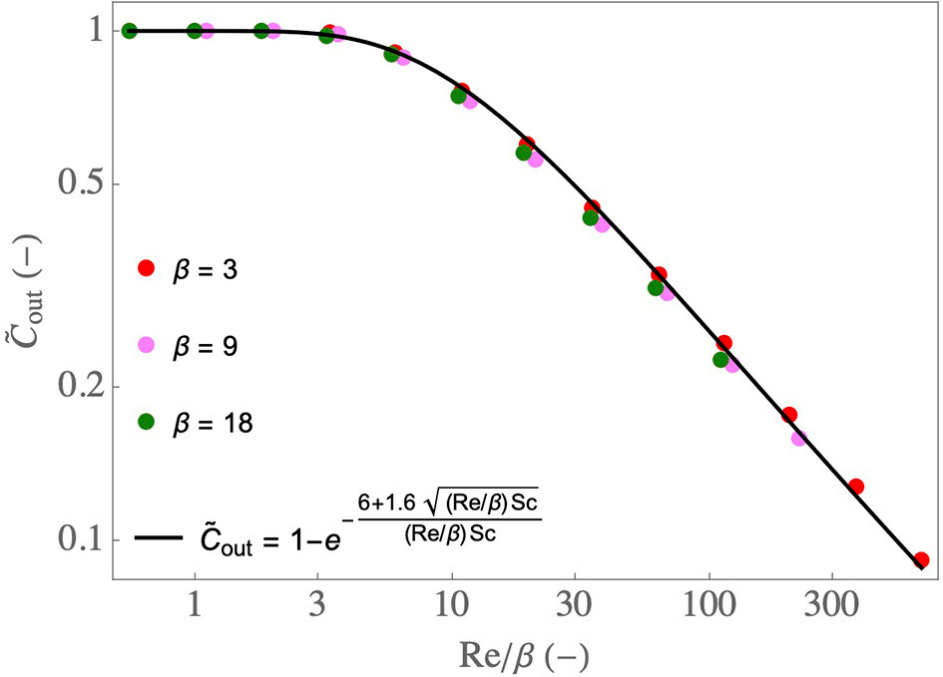
${\tilde{C}}_{\text{out}}$ as a function of Re/β, for various β, with ${\tilde{C}}_{\text{in}}=0$ and ${\tilde{C}}_{\mu }=1$. Dots: results of the CFD simulations of Supplementary Equations 3–13 in the Supplementary Material. Full line: results obtained using Equations (2), (3), and (4).

Equations (2)–(4) form together a simple model of the water transfer in the lumen of the airway. As described in the Supplementary Material, it is derived from the local transport equations using several assumptions, notably that *k* is constant in the airway. Therefore, to assess its quality, it is compared in Figure 4 with results of CFD simulations of the 2D continuity equation, momentum and water vapor transport equations, and their boundary conditions (Supplementary Equations 3–5, 7–13 in the Supplementary Material), for various values of Re and β, and with ${\tilde{C}}_{\text{in}}=0$ and ${\tilde{C}}_{\mu }=1$. An excellent agreement is observed between the CFD simulations and the simple model, validating therefore the latter. The results show that ${\tilde{C}}_{\text{out}}$ decreases if Re/β increases. It is a consequence of the fact that, if Re/β increases, βSh/Re decreases, and so does Ψ. Moreover, the results show that, if Re/β is smaller than 4–5, the airway is able to condition a dry air to the maximal possible extent (i.e., going from ${\tilde{C}}_{\text{in}}=0$ to ${\tilde{C}}_{\text{out}}={\tilde{C}}_{\mu }=1$).

As described in the Supplementary Material, an equation similar to Equation (2) can be established to describe the heat transfer in the lumen of the airway:

(5) $$\begin{array}{l} {\tilde{T}}_{\text{out}}=\frac{{\tilde{T}}_{\text{in}}-{\tilde{T}}_{\mu }}{\bar{\text{Ψ}}}+{\tilde{T}}_{\mu } \end{array}$$

with

(6) $$\begin{array}{l} \bar{\text{Ψ}}=\exp\left(4\beta \frac{\text{Nu}}{\text{Re Pr}}\right) \end{array}$$

${\tilde{T}}_{\text{in}}$ and ${\tilde{T}}_{\text{out}}$ are the averages of the dimensionless temperature on the flow cross-section at the inlet and at the outlet of the airway, respectively. ${\tilde{T}}_{\mu }$ is the dimensionless version of *T*_μ_. $\bar{\text{Ψ}}$ is the equivalent of Ψ regarding heat transfer and it involves two other dimensionless numbers: Nu and Pr. The Nusselt number Nu is a dimensionless heat transfer coefficient between the ASL and the lumen. It is defined as Nu = *uR*/λ, with *u* (W m^−2^ K^−1^) the heat transfer coefficient between the ASL and the lumen and λ (W m^−1^ K^−1^) the thermal conductivity of the air. Pr = ν/α is the Prandtl number of the air and α (m^2^ s^−1^) is the thermal diffusivity of the air. Pr is equal to 0.72 at body temperature and at a relative humidity of 100%.

A dimensional analysis of the local transport equations in the lumen of the airway and their boundary conditions show that, for a fixed value of Pr, Nu is a sole function of Re and of β. Therefore, as described in the Supplementary Material, CFD simulations of these equations can be used to construct a correlation relating Nu to Re and β: Nu = *g*(Re, β). As Pr and Sc are close to each other and as the heat and water vapor transport equations are similar, there is a strong similarity between heat and mass transfers in the lumen of the airway and, basically, Nu and Sh are quite close to each other (and so do Ψ and $\bar{\text{Ψ}}$). Hence, the whole above-mentioned conclusions drawn for water transfer can be transposed to heat transfer. Notably, the results show that, when Re/β > 1, Nu can be very well-approximated by Nu = $1.5+0.4\sqrt{\text{Re Pr}/\beta }$.

#### 2.1.2. Transfers in the Bronchial Wall and at the ASL–Lumen Interface

The equations developed in the previous section have to be completed by equations describing the transport phenomena in the bronchial wall and at the ASL–lumen interface (i.e., equations that will allow evaluating ${\tilde{C}}_{\mu }$ and ${\tilde{T}}_{\mu }$).

First of all, various phenomenological considerations must be introduced.

As sketched in Figure 2, the wall of the airway is composed of the ASL (thickness of about 10 μm) lining a layer of epithelial cells (thickness of about 10 μm) and surrounded by tissues of varying nature (lamina propria, muscles…). The heat transport in the wall of the airway is purely diffusive. Accordingly, an order of magnitude of the thickness of the wall of the airway affected by heat transfer is $\delta_{w}=\sqrt{\alpha_{w}t^{\text{insp}}}$, with α_*w*_ (m^2^ s^−1^) the thermal diffusivity of the tissues composing the airway wall. The value of δ_*w*_ for a human adult at rest (*t*^insp^ ≃ 2 s) is close to 500 μm. For all the results presented in this work, we have checked that $R\gg \sqrt{\alpha_{w}t^{\text{insp}}}$ in the airways with a significant contribution to heat and water exchanges (and, thus, we have also $L\gg \sqrt{\alpha_{w}t^{\text{insp}}}$, as β = *L*/*R* is always > 1). Moreover, as the temperature at the ASL–lumen interface, *T*_μ_, is assumed independent of the axial position in the airway, the same is assumed for the temperature in the bronchial wall. All these elements imply that the heat transport in the wall of the airway can be considered purely radial and described by a heat diffusion equation written in a 1D Cartesian axis system perpendicular to the airway wall (Supplementary Equation 28 in the Supplementary Material).

In addition, we assume that the tissues composing the wall of the airway are homogeneously vascularized by blood on a thickness larger than δ_*w*_. Using the data presented by Montaudon et al. (2007) regarding the vascularization of these tissues, we have checked that, for all the results presented in this work for a human adult, this assumption is valid in the airways with a significant contribution to heat and mass transfer. We introduce a characteristic time of the blood circulation in the tissues composing the wall of the airway, *t*_*w*_ (s), defined as the ratio of the volume of these tissues to the blood flow rate within them. For a human adult at rest, an order of magnitude of *t*_*w*_ can be evaluated. Indeed, at rest, the ratio of the volume of blood in the body to the cardiac flow rate is of the order of the minute. It can be assumed that $t_{w}^{\prime }$ (s), the ratio of the volume of blood in the tissues composing the wall of the airway to the blood flow rate within them is of the same order of magnitude. Moreover, in a tissue, the volume fraction of blood, ζ, is typically 2–4% (Hindel et al., 2017). Consequently, the order of magnitude of $t_{w}=t_{w}^{\prime }/\zeta$ is between 1,500 and 3,000 s. When an effort is realized, *t*_*w*_ is decreased (by a factor 2–4), as the cardiac flow rate is increased. Our representation of the bronchial wall and our estimation of *t*_*w*_ are supported by other works (Eisner and Martonen, 1989; McCullagh et al., 2010).

Having said that, an important feature of the dynamics of heat transport in the wall of the airway can be highlighted. First, as $t_{w}^{\prime }\gg t^{\text{insp}}$, the renewal of the blood in the wall of the airway takes place on a much larger time scale than the breathing time. Moreover, during inspiration, it can be easily evaluated that, per unit area of the ASL–lumen interface, the amount of heat that could be extracted by the ventilation from the wall of the airway, whose order of magnitude is $t^{\text{insp}}k\left(C_{\text{sat}}\left(T_{b}\right)-C_{0}\right)L_{m}$ (as mentioned in the introduction, the main cause of heat extraction from the mucosa is the energy used to evaporate water), with $L_{m}$ (J mol^−1^) the latent heat of vaporization of water, is much smaller than the amount of heat available in the wall, whose order of magnitude is $\sqrt{\alpha_{w}t^{\text{insp}}}\rho_{w}c_{p,w}\left(T_{b}-T_{0}\right)$, with ρ_*w*_ (kg m^−3^) and *c*_*p,w*_ (J kg^−1^ K^−1^) the density and the heat capacity of the tissues composing the airway wall, respectively. Consequently, during a single respiratory cycle, neither the renewal of blood by the circulation nor the extraction of heat by ventilation have the ability to significantly modify the bronchial wall temperature. In other words, we can assume that the temperature profile in the wall of the airway is at steady state (this assumption is consistent with the numerical results presented by Wu et al., 2014). This temperature profile is controlled by the time average, over a whole respiratory cycle, of the temperature and water concentration in the lumen of the airway.

Finally, we assume that there is no heat transfer limitation through the ASL and the epithelium, as they have a thickness much smaller than δ_*w*_.

According to these different elements and as presented in the Supplementary Material, a diffusion equation and its boundary conditions (Supplementary Equations 28–31 in the Supplementary Material) can be established to describe the heat transport in the wall of the airway. The resolution of this system of equations allows writing the following “interfacial” equation, in a dimensionless form:

(7) $$\begin{array}{l} \;\Lambda \left(1-{\tilde{T}}_{\mu }\right)=\frac{1}{1+\gamma }\left({\tilde{C}}_{\mu }-{\left[\tilde{C}\right]}^{\text{insp}}\right)+\frac{1}{1+\gamma }\Phi \left({\tilde{T}}_{\mu }-{\left[\tilde{T}\right]}^{\text{insp}}\right)\\ +\frac{\gamma }{1+\gamma }\frac{{\text{Sh}}^{\text{exp}}}{{\text{Sh}}^{\text{insp}}}\left({\tilde{C}}_{\mu }-{\left[\tilde{C}\right]}^{\text{exp}}\right)+\frac{\gamma }{1+\gamma }\Phi \frac{{\text{Nu}}^{\text{exp}}}{{\text{Nu}}^{\text{insp}}}\left({\tilde{T}}_{\mu }-{\left[\tilde{T}\right]}^{\text{exp}}\right) \end{array}$$

with

(8) $$\begin{array}{l} \Lambda =\frac{\lambda_{w}\left(T_{b}-T_{0}\right)}{L_{m}\frac{{\text{Sh}}^{\text{insp}}D}{R}\left(C_{\text{sat}}\left(T_{b}\right)-C_{0}\right)\sqrt{\alpha_{w}t_{w}}} \end{array}$$

(9) $$\begin{array}{l} \Phi =\frac{{\text{Nu}}^{\text{insp}}}{{\text{Sh}}^{\text{insp}}}\frac{\lambda }{DL_{m}}\frac{T_{b}-T_{0}}{C_{\text{sat}}\left(T_{b}\right)-C_{0}} \end{array}$$

In these equations, ${\left[\tilde{C}\right]}^{\text{insp}}$ and ${\left[\tilde{T}\right]}^{\text{insp}}$ are the averages of the dimensionless water concentration and temperature on the entire lumen of the airway during inspiration, while ${\left[\tilde{C}\right]}^{\text{exp}}$ and ${\left[\tilde{T}\right]}^{\text{exp}}$ are these averages during expiration. Sh^insp^ and Sh^exp^ are the values of the Sherwood number in the airway during inspiration and expiration, while Nu^insp^ and Nu^exp^ are the values of the Nusselt number in the airway during inspiration and expiration. λ_*w*_ (W m^−1^ K^−1^) is the thermal conductivity of the tissues composing the wall of the airway.

Equation (7) describes the competition between the heating of the tissues composing the airway wall by the flow of blood within them and the heat withdrawal from these tissues to condition the air. The left-hand side of the equation characterizes the ability of the vascularization to reheat the tissues if they are cooled by this conditioning (i.e., if ${\tilde{T}}_{\mu }<1$). On the other hand, the first two terms of the right-hand side of the equation characterize the heat withdrawal from the mucosa during inspiration, to evaporate water contained in the ASL and to heat the air in the lumen, while the two last terms characterizes this energy exchange during expiration.

The dimensionless number Λ compares the ability of the vascularization to reheat the tissues composing the wall of the airway and the intensity of the heat withdrawal to evaporate water in the ASL. It is clearly seen in Equation (7) that, if Λ ≫ 1, i.e., heat renewal by the vascularization dominates heat withdrawal, ${\tilde{T}}_{\mu }≃1$, i.e., the mucosa is at body temperature, and that, if Λ ≪ 1, i.e., heat withdrawal dominates heat renewal by the vascularization, the right-hand side of Equation (7) tends to zero, i.e., there is, on average during a breathing cycle, no transfer of energy between the mucosa and the air in the lumen.

Φ is a dimensionless number characterizing the relative importance of the two mechanisms in the air conditioning: the heating of the air up to body temperature and the saturation of the air with water. As Sh and Nu show similar dependence upon the Reynolds number, it appears that Φ is almost a constant (for given values of *C*_0_ and *T*_0_). The calculations presented in section 3 show that Φ is close to 0.15, for all the situations considered in this work (except when breathing very cold air). This is coherent with the calculation mentioned in the introduction, stating that the power used to evaporate water accounts for more than 80% of the total power extracted from the body by the ventilation.

Finally, assuming that the air in contact with the ASL is saturated with water (i.e., there is a local thermodynamic equilibrium at the ASL–lumen interface), the following dimensionless equation can be written to complete the description of the dynamics at the level of the mucosa:

(10) $$\begin{array}{l} {\tilde{C}}_{\mu }=\frac{C_{\text{sat}}\left({\tilde{T}}_{\mu }\left(T_{b}-T_{0}\right)+T_{0}\right)-C_{0}}{C_{\text{sat}}\left(T_{b}\right)-C_{0}} \end{array}$$

### 2.2. Heat and Water Exchanges in the Entire Bronchial Region of the Lungs

#### 2.2.1. Complete Model

The developments made at the level of a single airway can be generalized into a model of the heat and water exchanges in the *n* generations of the bronchial region of the lungs.

First, several notations should be precised. ${\tilde{C}}_{i}^{\text{insp}}$ and ${\tilde{T}}_{i}^{\text{insp}}$ are the averages of the dimensionless water concentration and temperature on the flow cross-section at the distal end (i.e., the farthest from the top of the trachea) of an airway in generation *i* during inspiration. ${\tilde{C}}_{0}^{\text{insp}}$ and ${\tilde{T}}_{0}^{\text{insp}}$ are the averages of the dimensionless water concentration and temperature of the air entering the trachea during inspiration. According to Equation (1):

(11) $$\begin{array}{l} {\tilde{C}}_{0}^{\text{insp}}={\tilde{T}}_{0}^{\text{insp}}=0 \end{array}$$

Similarly, ${\tilde{C}}_{i}^{\text{exp}}$ and ${\tilde{T}}_{i}^{\text{exp}}$ are the averages of the dimensionless water concentration and temperature on the flow cross-section at the proximal end (i.e., the closest from the top of the trachea) of an airway in generation *i* during expiration. ${\tilde{C}}_{n+1}^{\text{exp}}$ and ${\tilde{T}}_{n+1}^{\text{exp}}$ are the averages of the dimensionless water concentration and temperature of the air entering generation *n* during expiration. For all the conditions simulated in this paper, it appears that, during inspiration, the bronchial tract is able to fully condition the air (i.e., to bring it to body temperature and to saturate it with water). Hence, regarding heat and water exchanges, nothing happens in the alveolar region and, during expiration, the temperature of the air entering generation *n* is *T*_*b*_ and its water concentration is *C*_sat_(*T*_*b*_). Consequently:

(12) $$\begin{array}{l} {\tilde{C}}_{n+1}^{\text{exp}}={\tilde{T}}_{n+1}^{\text{exp}}=1 \end{array}$$

Finally, ${\tilde{C}}_{\mu ,i}$ and ${\tilde{T}}_{\mu ,i}$ are the dimensionless water vapor concentration and temperature at the ASL–lumen interface in an airway in generation *i*.

During inspiration and expiration, the Reynolds numbers of the flow in an airway in generation *i* are written ${\text{Re}}_{i}^{\text{insp}}$ and ${\text{Re}}_{i}^{\text{exp}}$, respectively. They can be calculated as:

(13) $$\begin{array}{l} {\text{Re}}_{i}^{\text{insp}}=\frac{2R_{i}}{\nu }\frac{Q^{\text{insp}}}{2^{i-1}\pi R_{i}^{2}}=\gamma {\text{Re}}_{i}^{\text{exp}} \end{array}$$

During inspiration and expiration, the Sherwood and Nusselt numbers in generation *i* are written ${\text{Sh}}_{i}^{\text{insp}}$, ${\text{Sh}}_{i}^{\text{exp}}$, ${\text{Nu}}_{i}^{\text{insp}}$, and ${\text{Nu}}_{i}^{\text{exp}}$, respectively. They are related to ${\text{Re}}_{i}^{\text{insp}}$, ${\text{Re}}_{i}^{\text{exp}}$, and β_*i*_ = *L*_*i*_/*R*_*i*_ using the correlations Sh = *f*(Re, β) and Nu = *g*(Re, β) obtained from the CFD simulations of the local transport equations. In generation *i* during inspiration and expiration, the dimensionless numbers Ψ and $\bar{\text{Ψ}}$ are written ${\text{Ψ}}_{i}^{\text{insp}}$, ${\text{Ψ}}_{i}^{\text{exp}}$, ${\bar{\text{Ψ}}}_{i}^{\text{insp}}$, and ${\bar{\text{Ψ}}}_{i}^{\text{exp}}$, respectively. They are evaluated using Equations (3) and (6).

Then, we can write the macroscopic mass and heat balance equations (Equations 2, 5) for each generation, for inspiration and expiration:

(14) $$\begin{array}{l} {\tilde{C}}_{i}^{\text{insp}}=\frac{{\tilde{C}}_{i-1}^{\text{insp}}-{\tilde{C}}_{\mu ,i}}{{\text{Ψ}}_{i}^{\text{insp}}}+{\tilde{C}}_{\mu ,i}\text{ and }{\tilde{T}}_{i}^{\text{insp}}=\frac{{\tilde{T}}_{i-1}^{\text{insp}}-{\tilde{T}}_{\mu ,i}}{{\bar{\text{Ψ}}}_{i}^{\text{insp}}}+{\tilde{T}}_{\mu ,i} \end{array}$$

(15) $$\begin{array}{l} {\tilde{C}}_{i}^{\text{exp}}=\frac{{\tilde{C}}_{i+1}^{\text{exp}}-{\tilde{C}}_{\mu ,i}}{{\text{Ψ}}_{i}^{\text{exp}}}+{\tilde{C}}_{\mu ,i}\text{ and }{\tilde{T}}_{i}^{\text{exp}}=\frac{{\tilde{T}}_{i+1}^{\text{exp}}-{\tilde{T}}_{\mu ,i}}{{\bar{\text{Ψ}}}_{i}^{\text{exp}}}+{\tilde{T}}_{\mu ,i} \end{array}$$

These 4*n* balance equations involve 6*n* unknowns (${\tilde{C}}_{i}^{\text{insp}}$, ${\tilde{T}}_{i}^{\text{insp}}$, ${\tilde{C}}_{i}^{\text{exp}}$, ${\tilde{T}}_{i}^{\text{exp}}$, ${\tilde{C}}_{\mu ,i}$, and ${\tilde{T}}_{\mu ,i}$, ∀*i* ∈ [1, *n*]). They are completed by the interfacial equations, Equations (7) and (10), written for each generation:

(16) $$\begin{array}{l} \Lambda_{i}\left(1-{\tilde{T}}_{\mu ,i}\right)=\frac{1}{1+\gamma }\left({\tilde{C}}_{\mu ,i}-{\left[{\tilde{C}}_{i}\right]}^{\text{insp}}\right)\\ +\frac{1}{1+\gamma }\Phi_{i}\left({\tilde{T}}_{\mu ,i}-{\left[{\tilde{T}}_{i}\right]}^{\text{insp}}\right)+\frac{\gamma }{1+\gamma }\frac{{\text{Sh}}_{i}^{\text{exp}}}{{\text{Sh}}_{i}^{\text{insp}}}\left({\tilde{C}}_{\mu ,i}-{\left[{\tilde{C}}_{i}\right]}^{\text{exp}}\right)\\ +\frac{\gamma }{1+\gamma }\Phi_{i}\frac{{\text{Nu}}_{i}^{\text{exp}}}{{\text{Nu}}_{i}^{\text{insp}}}\left({\tilde{T}}_{\mu ,i}-{\left[{\tilde{T}}_{i}\right]}^{\text{exp}}\right) \end{array}$$

(17) $$\begin{array}{l} {\tilde{C}}_{\mu ,i}=\frac{C_{\text{sat}}\left({\tilde{T}}_{\mu ,i}\left(T_{b}-T_{0}\right)+T_{0}\right)-C_{0}}{C_{\text{sat}}\left(T_{b}\right)-C_{0}} \end{array}$$

with Λ_*i*_ and Φ_*i*_ the values of Λ (see Equation 8) and Φ (see Equation 9) in generation *i*. ${\left[{\tilde{C}}_{i}\right]}^{\text{insp}}$, ${\left[{\tilde{T}}_{i}\right]}^{\text{insp}}$, ${\left[{\tilde{C}}_{i}\right]}^{\text{exp}}$, and ${\left[{\tilde{T}}_{i}\right]}^{\text{exp}}$ are the values of ${\left[\tilde{C}\right]}^{\text{insp}}$, ${\left[\tilde{T}\right]}^{\text{insp}}$, ${\left[\tilde{C}\right]}^{\text{exp}}$, and ${\left[\tilde{T}\right]}^{\text{exp}}$ in generation *i*. We propose to express each of them as ${\left[{ỹ}_{i}\right]}^{\text{insp}}=\left({ỹ}_{i}^{\text{insp}}+{ỹ}_{i-1}^{\text{insp}}\right)/2$ and ${\left[{ỹ}_{i}\right]}^{\text{exp}}=\left({ỹ}_{i}^{\text{exp}}+{ỹ}_{i+1}^{\text{exp}}\right)/2$, respectively, with *y* equal to *C* or *T*.

#### 2.2.2. Simplified Framework

To highlight the key phenomena and dimensionless numbers governing the exchanges of heat and water in human lungs of various sizes, it is interesting to place ourselves in a simplified framework, described in this section. The results obtained with this simplified framework are presented in the first part of section 3 (section 3.1).

First, we use a simplified representation of the lungs, where *R*_*i*+1_ = *hR*_*i*_, with *h* = 2^−1/3^, and *L*_*i*_/*R*_*i*_ = β (independent of *i*), as in Mauroy et al. (2004), Noel and Mauroy (2019), and West et al. (1997). To calculate the number *n* of generations in the bronchial tree, we assume that this tree stops when the successive divisions have generated airways with a radius smaller than the alveolar diameter, *d*_alv_ (i.e., $h^{n-1}R_{1}<d_{\text{alv}}$ and $h^{n-2}R_{1}>d_{\text{alv}}$). Such a geometry is entirely specified by the values of *R*_1_, β and *d*_alv_. To give a broad overview, these parameters are expressed as functions of the body mass, *M* (kg), using allometric laws presented by West et al. (1997) and scaled using data given in Karamaoun et al. (2016): $R_{1}\propto M^{3/8}$, with a proportionality coefficient such that *R*_1_ = *R*_1,ref_ = 7.5 mm for a reference mass *M*_ref_ = 70 kg, β ∝ *M*^−1/8^, with a proportionality coefficient such that β = β_ref_ = 7 for *M* = *M*_ref_, and $d_{\text{alv}}\propto M^{1/12}$, with a proportionality coefficient such that *d*_alv_ = *d*_alv,ref_ = 200 μm for *M* = *M*_ref_.

Two important parameters of the problem, the inspiration flow rate, *Q*^insp^, and the characteristic time of blood renewal in the tissues surrounding the wall of an airway, *t*_*w*_, can also be expressed as functions of *M* (West et al., 1997; Karamaoun et al., 2018):

(18) $$\begin{array}{l} Q^{\text{insp}}=\psi Q_{\text{ref}}^{\text{insp}}{\left(\frac{M}{M_{\text{ref}}}\right)}^{3/4} \end{array}$$

(19) $$\begin{array}{l} t_{w}=\frac{t_{w,\text{ref}}}{\phi }{\left(\frac{M}{M_{\text{ref}}}\right)}^{1/4} \end{array}$$

with $Q_{\text{ref}}^{\text{insp}}=15$ l min^−1^ and *t*_*w*,ref_ = 2, 000 s (see section 2.1.2). ψ and ϕ are factors accounting for the increase of the inspiration flow rate and the cardiac flow rate during a possible effort. ψ = 1 at rest and it can reach values up to 8–10 during an intense effort. ϕ = 1 at rest and it can reach values up to 3–4 during an intense effort.

Due to the simplified geometry used, the Reynolds numbers of the flow in an airway in generation *i*, during inspiration and expiration, can be expressed as functions of *i* and of the Reynolds number of the flow in the trachea during inspiration, ${\text{Re}}_{1}^{\text{insp}}$:

(20) $$\begin{array}{l} \gamma {\text{Re}}_{i}^{\text{exp}}={\text{Re}}_{i}^{\text{insp}}=\frac{2R_{i}}{\nu }\frac{Q^{\text{insp}}}{2^{i-1}\pi \;R_{i}^{2}}=\frac{2R_{1}}{\nu }\frac{Q^{\text{insp}}}{{\left(2h\right)}^{i-1}\pi \;R_{1}^{2}}=\frac{{\text{Re}}_{1}^{\text{insp}}}{{\left(2h\right)}^{i-1}} \end{array}$$

As *h* = 2^−1/3^, the Reynolds numbers decrease by a factor 4 when we progress 3 generations in the bronchial tree.

In this simplified framework, we also use the correlation Sh = $1.5+0.4\sqrt{\text{Sc Re}/\beta }$ (Equation 4), giving a very good approximation of the numerical results when Re/β > 1 (see Figure 3A). It characterizes the transition regime of mass transfer for low values of Re/β (as it gives Sh ≃ 1.5) and the boundary layer regime for large values of Re/β. For the results presented in this paper, the condition Re/β > 1 is fullfilled in all the airways with a significant contribution to water exchanges (i.e., variations of the dimensionless temperature and water concentration larger than 1%). Introducing this expression of Sh in the equation giving Ψ (Equation 3) and using Equation (20), an analytical expression of ${\text{Ψ}}_{i}^{\text{insp}}$, the dimensionless number in the mass balance equation for the inspiration (Equation 14), can be obtained:

(21) $$\begin{array}{l} {\text{Ψ}}_{i}^{\text{insp}}=\exp\left(\frac{6\beta {\left(2h\right)}^{i-1}}{{\text{Re}}_{1}^{\text{insp}}\text{Sc}}\right)\exp\left(1.6\sqrt{\frac{\beta {\left(2h\right)}^{i-1}}{{\text{Re}}_{1}^{\text{insp}}\text{Sc}}}\right) \end{array}$$

A similar expression can be obtained for ${\text{Ψ}}_{i}^{\text{exp}}$.

We finally further simplify the framework by linearizing Equation (17) with respect to ${\tilde{T}}_{\mu ,i}$ (around ${\tilde{T}}_{\mu ,i}=1$), eliminating ${\tilde{T}}_{\mu ,i}$ between this linearized equation and Equation (16), and taking the limit Φ_*i*_ → 0 (i.e., neglecting the amount of heat extracted from the mucosa to heat the air). This yields the following linear equation replacing Equation (16):

(22) $$\begin{array}{r} \Lambda_{i}^{\prime }\left(1-{\tilde{C}}_{\mu ,i}\right)=\frac{1}{1+\gamma }\left({\tilde{C}}_{\mu ,i}-{\left[{\tilde{C}}_{i}\right]}^{\text{insp}}\right)\\ +\frac{\gamma }{1+\gamma }\frac{{\text{Sh}}_{i}^{\text{exp}}}{{\text{Sh}}_{i}^{\text{insp}}}\left({\tilde{C}}_{\mu ,i}-{\left[{\tilde{C}}_{i}\right]}^{\text{exp}}\right) \end{array}$$

with, according to the different elements introduced previously:

(23) $$\begin{array}{l} \Lambda_{i}^{\prime }=\Theta \sqrt{\frac{\phi }{\psi }}\frac{1}{1+3.75\sqrt{\frac{\beta {\left(2h\right)}^{i-1}}{{\text{Re}}_{1}^{\text{insp}}\text{Sc}}}} \end{array}$$

a dimensionless number with the same physical meaning than Λ_*i*_. It is a function of *i*, ${\text{Re}}_{1}^{\text{insp}}/\beta$ and ϕ/ψ. Θ is a constant (≃ 0.5 at atmospheric pressure) given by:

(24) $$\begin{array}{l} \Theta =\frac{\lambda_{w}R_{1,\text{ref}}^{3/2}}{0.4L_{m}D\sqrt{\alpha_{w}}\frac{dC_{\text{sat}}}{dT}{|}_{T=T_{b}}}\sqrt{\frac{\pi \;\nu \beta_{\text{ref}}}{2Q_{\text{ref}}^{\text{insp}}t_{w,\text{ref}}\text{Sc}}} \end{array}$$

It is worth noting that Equation (22), simplified version of Equation (16), does not involve the temperature. Therefore, with the boundary conditions (11)–(12) and the mass balances given in Equations (14)–(15), it offers the possibility to calculate the dimensionless water concentration profiles in the bronchial region of the lungs, during inspiration and expiration, without the need of calculating the temperature profiles. This simplified dimensionless model involves solely five dimensionless numbers, independent of the environmental conditions: Sc and Θ, both having fixed values, ${\text{Re}}_{1}^{\text{insp}}/\beta$, depending on the size of the body and on the intensity of a possible effort, γ, the ratio of the expiration time to the inspiration time and ϕ/ψ, the ratio of the cardiac flow rate increase to the ventilation rate increase during an effort. Results provided in the Supplementary Material show that this simplified model gives actually a very good estimation of the dimensionless water vapor concentration profiles in the lungs (and consequently of all the related dimensionless parameters) when it is compared to the complete model on the same geometry.

This simplified framework only considers the water exchanges in the lungs. However, as pointed out in the introduction and shown in the Supplementary Material, since most of the energy extracted from the lungs is used to evaporate water contained in ASL, there is a direct parallel between the exchanges of heat and water in the lungs. Thus, qualitatively, all the comments elaborated in section 3.1 when analyzing the results obtained with this simplified model can be transposed to the heat exchanges within the lungs.

#### 2.2.3. Summary of the Models and Post-processing of the Calculation Results

**Figure 5:**
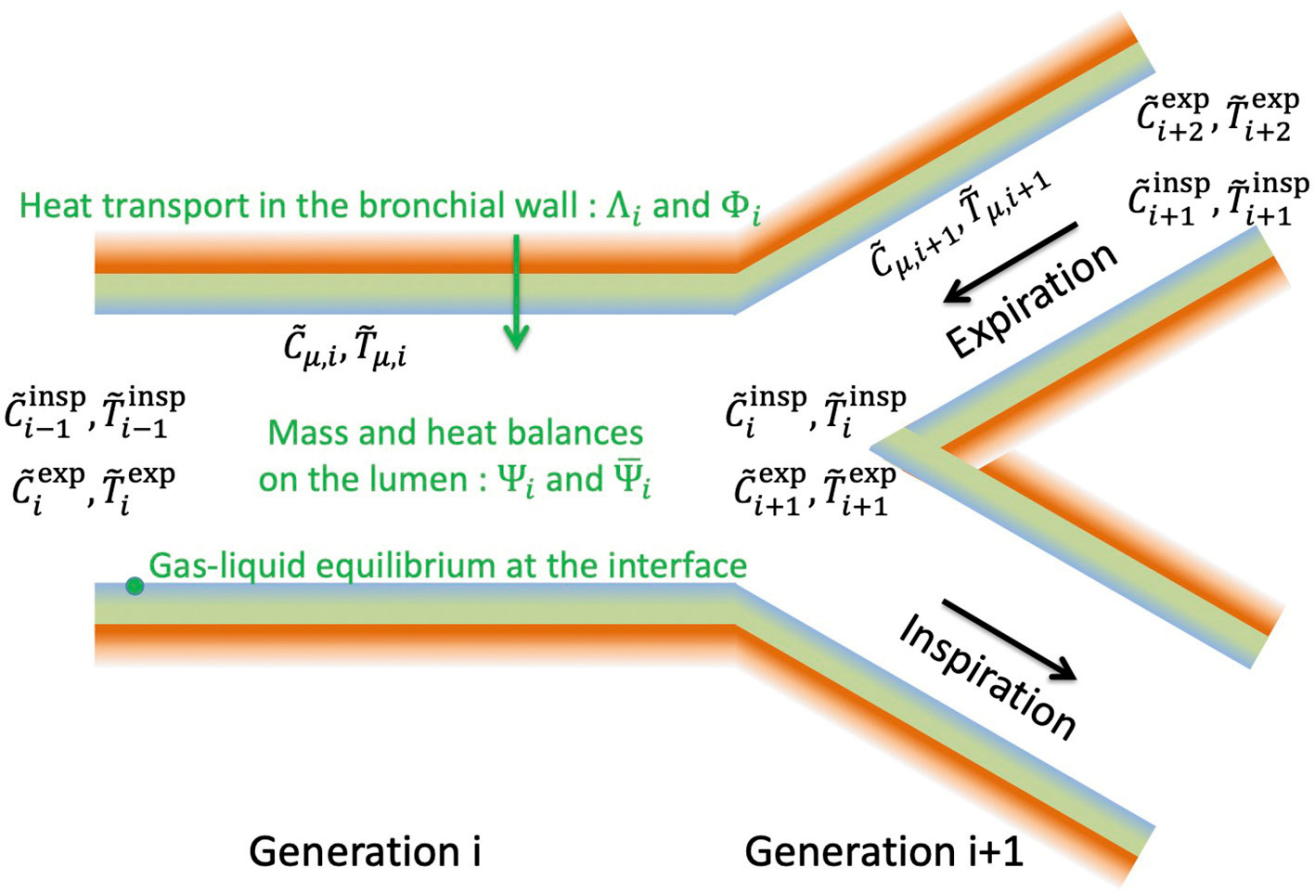
General structure of the model developed in this paper.

The models (complete and simplified) developed in this paper are summarized in Figure 5 and the different dimensionless numbers involved are described in Table 1.

In generation *i*, the heat transfers in the bronchial wall and at the ASL–Lumen interface are modeled by Equations (16)–(17), involving the two dimensionless numbers Λ_*i*_ and Φ_*i*_. These numbers largely influence the value of the temperature of the ASL, ${\tilde{T}}_{\mu ,i}$, and hence the water concentration in the air in contact with the ASL, ${\tilde{C}}_{\mu ,i}$, since a local equilibrium is assumed achieved at the ASL–lumen interface (Equation 17).

During inspiration, the water concentration and temperature in the lumen at the entrance of generation *i* are ${\tilde{C}}_{i-1}^{\text{insp}}$ and ${\tilde{T}}_{i-1}^{\text{insp}}$, respectively. At the outlet of the generation, the water concentration and temperature in the lumen, ${\tilde{C}}_{i}^{\text{insp}}$ and ${\tilde{T}}_{i}^{\text{insp}}$, are linked to ${\tilde{C}}_{i-1}^{\text{insp}}$, ${\tilde{T}}_{i-1}^{\text{insp}}$, ${\tilde{C}}_{\mu ,i}$, and ${\tilde{T}}_{\mu ,i}$ by expressing mass and heat balances (Equation 14), involving the dimensionless numbers ${\text{Ψ}}_{i}^{\text{insp}}$ and ${\bar{\text{Ψ}}}_{i}^{\text{insp}}$, characterizing the ability of the transport phenomena within the lumen to condition the air. During inspiration, the dimensionless water concentration and temperature at the inlet of the first generation are set to zero (Equation 11).

During expiration, the water concentration and temperature in the lumen at the outlet of the generation *i*, ${\tilde{C}}_{i}^{\text{exp}}$, and ${\tilde{T}}_{i}^{\text{exp}}$, are linked to ${\tilde{C}}_{i+1}^{\text{exp}}$, ${\tilde{T}}_{i+1}^{\text{exp}}$ (their values at the inlet), ${\tilde{C}}_{\mu ,i}$ and ${\tilde{T}}_{\mu ,i}$ by also expressing mass and heat balances (Equation 15). At the inlet of generation *n* during expiration, we assume that the dimensionless water concentration and temperature are equal to 1 (Equation 12).

Inspiration and expiration are coupled by Equation (16), arising from the assumption that the temperature profile in the bronchial wall is at stationnary state. So, all the equations of the model (6 equations per generation) have to be solved simultaneously.

As mentioned previously, in the simplified framework, Equation (17) is linearized and subsequently combined with Equation (16) to yield Equation (22). It allows decoupling mass and heat transfers and, hence, 4*n* linear equations have to be solved. In this simplified framework, the dimensionless number Λ is replaced by Λ′ (see Equation 23). Moreover, a particular geometrical representation of the lungs is used in the simplified framework, while it remains general in the complete model (i.e., the latter can be used with any set of values of *L*_*i*_, *R*_*i*_, and *n*).

The solution of the models (complete and simplified) provide the concentration and temperature profiles in the bronchial region of the lungs during a respiratory cycle (temperature profile: only with the complete model). From that, several interesting quantities can be calculated:

- The amount of water extracted from the mucosa in generation *i* per unit of time:
  (25) $$\begin{array}{l} W_{i}=\frac{Q^{\text{insp}}}{1+\gamma }\left(C_{\text{sat}}\left(T_{b}\right)-C_{0}\right)\left({\tilde{C}}_{i}^{\text{insp}}-{\tilde{C}}_{i-1}^{\text{insp}}+{\tilde{C}}_{i}^{\text{exp}}-{\tilde{C}}_{i+1}^{\text{exp}}\right) \end{array}$$
- The total amount of water extracted per unit of time from the lungs by the ventilation:
  (26) $$\begin{array}{l} W=\overset{i=n}{\underset{i=1}{\sum }}W_{i}=\frac{Q^{\text{insp}}}{1+\gamma }\left(C_{\text{sat}}\left(T_{b}\right)-C_{0}\right)\;{\tilde{C}}_{1}^{\text{exp}} \end{array}$$
  This expression is obtained by assuming that ${\tilde{C}}_{n}^{\text{insp}}=1$ (and using the boundary conditions 12–13). This is supported by the calculation results, as they show that the air is fully conditioned in the bronchial tract during inspiration in all the cases considered in this work.
- The local efficiency of water extraction in generation *i*, defined as:
  (27) $$\begin{array}{l} \eta_{i}=\frac{\left({\tilde{C}}_{i}^{\text{insp}}-{\tilde{C}}_{i-1}^{\text{insp}}\right)+\left({\tilde{C}}_{i}^{\text{exp}}-{\tilde{C}}_{i+1}^{\text{exp}}\right)}{{\tilde{C}}_{i}^{\text{insp}}-{\tilde{C}}_{i-1}^{\text{insp}}} \end{array}$$
  It is the ratio of the amount of water extracted from the mucosa in generation *i* during a whole respiratory cycle to the amount of water extracted in this generation during inspiration only. 0 < η_*i*_ < 1 because the mucosa is cooled during inspiration (i.e., *T*_μ, *i*_ < 1) and, hence, condensation occurs during expiration as the air originating from the alveolar region is at body temperature and saturated with water. η_*i*_ ≃ 1 means almost no condensation during expiration while η_*i*_ ≃ 0 means that the water extracted from the mucosa during inspiration is almost totally given back during expiration.
- The overall efficiency of water extraction from the lungs:
  (28) $$\begin{array}{l} \eta =\frac{W}{W_{\text{max}}}={\tilde{C}}_{1}^{\text{exp}} \end{array}$$
  It is defined as the ratio of *W* to its maximal value, *W*_max_, obtained for ${\tilde{C}}_{1}^{\text{exp}}=1$ (no condensation during expiration).
- The time average of the evaporation rate in an airway of generation *i*:
  (29) $$\begin{array}{l} E_{i}=\frac{W_{i}}{\left(2^{i-1}2\pi \;R_{i}L_{i}\right)} \end{array}$$
- The total power extracted from the lungs by the ventilation:
  (30) $$\begin{array}{l} P=\frac{Q^{\text{insp}}}{1+\gamma }\left(\rho c_{p}\left(T_{b}-T_{0}\right){\tilde{T}}_{1}^{\text{exp}}+L_{m}\left(C_{\text{sat}}\left(T_{b}\right)-C_{0}\right){\tilde{C}}_{1}^{\text{exp}}\right) \end{array}$$
- The overall efficiency of heat extraction from the lungs:
  (31) $$\begin{array}{l} \bar{\eta }=\frac{P}{P_{\text{max}}} \end{array}$$
  It is defined as the ratio of *P* to its maximal value, *P*_max_, obtained for ${\tilde{C}}_{1}^{\text{exp}}={\tilde{T}}_{1}^{\text{exp}}=1$.

Note that, in the following, *W*_*i*_, *W*, and *W*_max_ are expressed as a volume of liquid water per unit of time.

## 3. Results and Discussion

### 3.1. Key Phenomena and Scaling Laws

To highlight the key phenomena and dimensionless numbers governing the exchanges of heat and water in lungs of various size, we use in this section the simplified framework described in section 2.2.2. The typical value of 2 for γ is taken but the dimensionless results presented here show no significant difference with those obtained with γ = 1. Consequently, ${\text{Re}}_{1}^{\text{insp}}/\beta$ and ϕ/ψ are the only parameters that are left variable. According to the relations stated above, ${\text{Re}}_{1}^{\text{insp}}/\beta$ is an increasing function of the mass of the body. For instance, we have, at rest (ψ = 1), ${\text{Re}}_{1}^{\text{insp}}/\beta ≃40$ for a newborn (*M* = 3 kg), ${\text{Re}}_{1}^{\text{insp}}/\beta ≃80$ for a young child (*M* = 15 kg), ${\text{Re}}_{1}^{\text{insp}}/\beta ≃150$ for a small adult (*M* = 50 kg) and ${\text{Re}}_{1}^{\text{insp}}/\beta ≃260$ for a large adult (*M* = 150 kg). During an effort, ${\text{Re}}_{1}^{\text{insp}}/\beta$ can reach values up to 1,000–1,500 for an adult.

In the following, when a quantity of interest is plotted as a function of the generation index *i*, the plot is limited to generations in which Re/β > 1, in order for the approximate expression of Sh as a function of Re/β to be valid (Equation 4).

**Figure 6:**
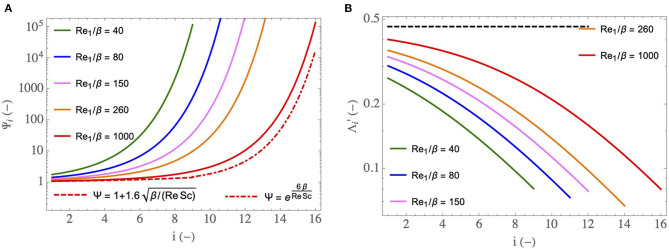
**(A)** ${\text{Ψ}}_{i}^{\text{insp}}$ as a function of *i*, for different ${\text{Re}}_{1}^{\text{insp}}/\beta$. **(B)** $\Lambda_{i}^{\prime }$ as a function of *i*, for different ${\text{Re}}_{1}^{\text{insp}}/\beta$ and for ϕ/ψ = 1. The dashed curve is the limit of $\Lambda_{i}^{\prime }$ for ${\text{Re}}_{1}^{\text{insp}}/\beta \to \infty$ and ϕ/ψ = 1 (i.e., it is the value of Θ). ${\text{Re}}_{1}^{\text{insp}}/\beta =40$ corresponds to a newborn at rest (*M* = 3 kg), ${\text{Re}}_{1}^{\text{insp}}/\beta =80$ to a young child at rest (*M* = 15 kg), ${\text{Re}}_{1}^{\text{insp}}/\beta =150$ to a small adult at rest (*M* = 50 kg), ${\text{Re}}_{1}^{\text{insp}}/\beta =260$ to a large adult at rest (*M* = 150 kg) and ${\text{Re}}_{1}^{\text{insp}}/\beta =1,000$ to an adult exercising.

First, in Figure 6, we show how two key dimensionless numbers depend on *i* and on ${\text{Re}}_{1}^{\text{insp}}/\beta$. In these figures, the superscript “insp” has been omitted for sake of clarity.

In Figure 6A, the dimensionless number ${\text{Ψ}}_{i}^{\text{insp}}$ (see Equation 21), characterizing the ability of the transport phenomena within the lumen of an airway in generation *i* to condition the air during inspiration, is plotted as a function of *i*, for five values of ${\text{Re}}_{1}^{\text{insp}}/\beta$. We have seen previously that Ψ decreases with an increase of Re/β. Consequently, ${\text{Ψ}}_{i}^{\text{insp}}$ decreases with an increase of ${\text{Re}}_{1}^{\text{insp}}/\beta$ and increases with *i*. The values of ${\text{Ψ}}_{i}^{\text{insp}}$ obtained in the distal generations are much larger than one. It shows that the smallest airways are “super-conditioners”. The decrease of ${\text{Ψ}}_{i}^{\text{insp}}$ with an increase of ${\text{Re}}_{1}^{\text{insp}}/\beta$ indicates that the transport phenomena within the airways of a newborn have a better ability to condition the air than those in the airways of an adult. It also indicates that this ability decreases during an effort.

The expression of ${\text{Ψ}}_{i}^{\text{insp}}$ can be simplified in two limit situations. For ${\text{Re}}_{1}^{\text{insp}}/\left(\beta {\left(2h\right)}^{i-1}\right)\gg 1$ (i.e., if we are in the boundary layer regime of water transfer), a Taylor expansion of Equation (21) allows writing ${\text{Ψ}}_{i}^{\text{insp}}≃1+1.6{\left({\left(2h\right)}^{i-1}\beta /\left({\text{Re}}_{1}^{\text{insp}}\text{Sc}\right)\right)}^{1/2}$. This relation is represented by the dashed curve in Figure 6A, for ${\text{Re}}_{1}^{\text{insp}}/\beta =1,000$. We see that this curve matches the corresponding full line curve in the proximal generations, thus marking the presence of the boundary layer regime of water transfer in these generations. On the other hand, if ${\text{Re}}_{1}^{\text{insp}}/\left(\beta {\left(2h\right)}^{i-1}\right)$ is small (i.e., if we are in the transition regime of water transfer), ${\text{Ψ}}_{i}^{\text{insp}}≃\exp\left(6\beta {\left(2h\right)}^{i-1}/\left({\text{Re}}_{1}^{\text{insp}}\text{Sc}\right)\right)$. This relation is dashed-dotted plotted in Figure 6A, for ${\text{Re}}_{1}^{\text{insp}}/\beta =1,000$. We see that this curve matches rather well the corresponding full line curve in distal generations, thus marking the presence, within the lungs, of several generations in which the regime of water transfer between the ASL and the lumen is the transition one. Moreover, we can see that, within this transition regime, ${\text{Ψ}}_{i}^{\text{insp}}$ increases faster than exponentially with *i*. For the sake of clarity, the dashed and dashed-dotted curves are plotted here for ${\text{Re}}_{1}^{\text{insp}}/\beta =1,000$ only, but same observations are drawn for the other values of ${\text{Re}}_{1}^{\text{insp}}/\beta$. However, the transition between the boundary layer regime and the transition one takes place closer and closer to the trachea as ${\text{Re}}_{1}^{\text{insp}}/\beta$ decreases. For a newborn (${\text{Re}}_{1}^{\text{insp}}/\beta =40$), the transition regime is met in almost all the bronchial region of the lungs.

In Figure 6B, another important dimensionless number, $\Lambda_{i}^{\prime }$ (see Equation 23), is plotted as a function of *i*, for several values of ${\text{Re}}_{1}^{\text{insp}}/\beta$ and for ϕ/ψ = 1. $\Lambda_{i}^{\prime }$ compares the ability of the vascularization to reheat the tissues composing the wall of an airway and the intensity of the heat withdrawal to evaporate water in the ASL. It increases with ${\text{Re}}_{1}^{\text{insp}}/\beta$ and decreases with an increase of *i*. We see in Figure 6B that, when ϕ/ψ = 1, $\Lambda_{i}^{\prime }$ is between 0.05 and 0.4. This implies that, at rest, a characteristic time of heat supply by the vascularization is between 2.5 and 20 times larger than a characteristic time of heat withdrawal to evaporate water contained in the ASL. This explains the results obtained by Mcfadden et al. (1985) and mentioned in the introduction: during inspiration, the mucosa is markedly cooled as the vascularization is unable to offset the energy extraction. Consequently, a significant cooling of the air and condensation of water occur during expiration.

In Figure 6B, the dashed curve is the limit of $\Lambda_{i}^{\prime }$ for ${\text{Re}}_{1}^{\text{insp}}/\beta \to \infty$ (and for ϕ/ψ = 1). According to Equation (23), this limit is independent of the generation index and is equal to the constant Θ (≃ 0.5). We see in Figure 6B that this limit is only approached in the first generations and for the highest considered value of ${\text{Re}}_{1}^{\text{insp}}/\beta$.

During an effort, the ratio ϕ/ψ usually takes values smaller than one since the ventilation rate increases more than the cardiac flow rate. As mentioned previously, ϕ can reach values up to 3–4 during an intense effort while ψ can reach values up to 8–10. Consequently, the range of possible values of ϕ/ψ is approximately 0.3–1. Thus, we see in Equation (23) that $\Lambda_{i}^{\prime }$ is reduced during an effort, and can go until being multiplied by a factor $\sqrt{0.3}≃0.55$. This is logical since, if the cardiac output increases less than the ventilation flow rate, the heating of the mucosa by the vascularization is disadvantaged compared to the extraction of heat from it by the evaporation. Consequently, air cooling and water condensation on expiration are favored when an effort is made.

**Figure 7:**
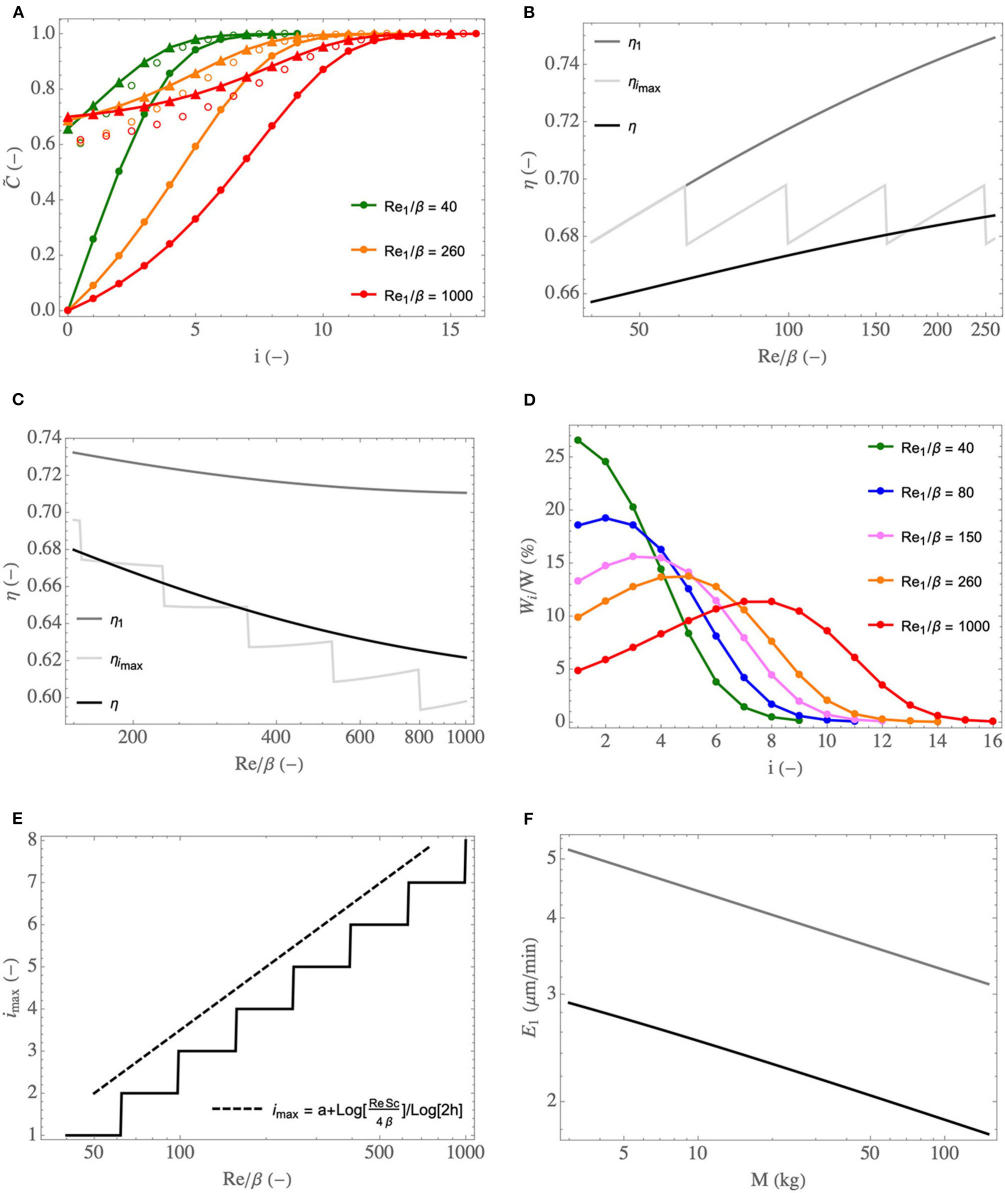
**(A)** Dimensionless water concentration profiles in the lumen, during inspiration (full circles) and expiration (triangles), for different ${\text{Re}}_{1}^{\text{insp}}/\beta$ and for ϕ/ψ = 1. Empty circles: values of ${\tilde{C}}_{\mu ,i}$. **(B)** η, η_1_, and η_*i*_max__ as functions of ${\text{Re}}_{1}^{\text{insp}}/\beta$, for ϕ/ψ = 1. **(C)** idem but with ϕ/ψ progressively decreasing with an increase of ${\text{Re}}_{1}^{\text{insp}}/\beta$, to simulate an increasing effort. **(D)** *W*_*i*_/*W* as a function of *i*, for different ${\text{Re}}_{1}^{\text{insp}}/\beta$ and for ϕ/ψ = 1. **(E)** Index of the generation in which the maximal amount of water is extracted as a function of ${\text{Re}}_{1}^{\text{insp}}/\beta$ and for ϕ/ψ = 1 (full line curve), and the scaling law (dashed curve). **(F)** *E*_1_ as a function of *M*, at rest (black curve) and during an effort (gray curve, calculated with ψ = 6 and ϕ = 2).

The analysis of the key phenomena related to heat and water exchanges in the lungs is further developed in Figure 7, where we show how some global or local quantities of interest, still calculated by solving the equations of the model in the simplified framework, depend on ${\text{Re}}_{1}^{\text{insp}}/\beta$, on the generation index, *i*, or on the mass of the body, *M*. In these figures, the superscript “insp” has been omitted for sake of clarity.

In Figure 7A, the profiles of the dimensionless water vapor concentration in the lumen, during inspiration and expiration, are presented for three values of ${\text{Re}}_{1}^{\text{insp}}/\beta$ (corresponding roughly to a newborn at rest, to an adult at rest and to an adult exercising). ϕ/ψ = 1 is used but, actually, these profiles are quite insensitive to ϕ/ψ (within the range of its possible values, approximately 0.3–1). Several elements, consistent with what is mentioned above, can be observed in this figure. First, we see clearly that the bronchial tract is able to condition the air fully, in the three situations considered. Second, a significant condensation during expiration is indeed predicted by the model. This is a consequence of the low values of $\Lambda_{i}^{\prime }$ compared to 1, which indicate an inability of the vascularization of the tissues surrounding the airways to offset their cooling induced by evaporation during inspiration. We also see that the bronchi of the newborn need less generations to condition the air. Even if the values of $\Lambda_{i}^{\prime }$ are lower for a newborn than for an adult (see Figure 6B), which is not in favor of air conditioning because a decrease of $\Lambda_{i}^{\prime }$ indicates a decrease in the capacity of the vascularization to maintain the tissues at body temperature, this is more than offset by the fact that the coefficients ${\text{Ψ}}_{i}^{\text{insp}}$ are larger for a newborn than for an adult (see Figure 6A). Finally, we also see that, during an effort, due to the increased flow rate of air in the lungs and the subsequent decrease of ${\text{Ψ}}_{i}^{\text{insp}}$, the air conditioning involves a larger number of generations than at rest.

In Figure 7B, the black curve gives the overall efficiency of water extraction from the lungs by the ventilation, η (see Equation 28), as a function of ${\text{Re}}_{1}^{\text{insp}}/\beta$, the gray curve gives the value of the local efficiency of water extraction in the trachea, η_1_ (see Equation 27), and the light gray curve gives the value of the local efficiency in the generation *i*_max_ in which the maximal amount of water is extracted, η_*i*_max__. ϕ = ψ = 1 is used (rest situation) and the range ${\text{Re}}_{1}^{\text{insp}}/\beta =40$ (a newborn) to ${\text{Re}}_{1}^{\text{insp}}/\beta =260$ (a large adult) is considered. In Figure 7C, η, η_1_, and η_*i*_max__ are also plotted as a function of ${\text{Re}}_{1}^{\text{insp}}/\beta$ but, here, the values of ϕ and ψ are linearly related to ${\text{Re}}_{1}^{\text{insp}}/\beta$: $\psi =\left({\text{Re}}_{1}^{\text{insp}}/\beta \right)/150$ and $\phi =0.7+0.002{\text{Re}}_{1}^{\text{insp}}/\beta$. This allows simulating the increasing effort of a single person, with a rest situation corresponding to ${\text{Re}}_{1}^{\text{insp}}/\beta =150$ (because it gives ϕ = ψ = 1) and with a cardiac output multiplied by 2.5 when the ventilation rate is multiplied by 6 (i.e., when ${\text{Re}}_{1}^{\text{insp}}/\beta =900$). We see in these figures that η, η_1_ and η_*i*_max__ experience small variations over the ranges of Re^insp^/β considered. At rest (Figure 7B), η and η_1_ slightly increase with ${\text{Re}}_{1}^{\text{insp}}/\beta$. This was expected as we understand that η and η_1_ are mostly controlled by the values of the dimensionless numbers $\Lambda_{i}^{\prime }$; $\Lambda_{i}^{\prime }$ much larger than 1 would lead to an efficiency of water extraction close to 1, while $\Lambda_{i}^{\prime }$ much smaller than 1 would lead to an efficiency close to 0. And, at a constant value of ϕ/ψ, $\Lambda_{i}^{\prime }$ increases with ${\text{Re}}_{1}^{\text{insp}}/\beta$ (see Figure 6B). On the other hand, we see in Figure 7C that η and η_1_ decrease slightly with the intensity of the effort. It is because the influence of ϕ/ψ on $\Lambda_{i}^{\prime }$ (decrease of $\Lambda_{i}^{\prime }$ with a decrease of ϕ/ψ, i.e., with the intensity of an effort) overwhelms the influence of ${\text{Re}}_{1}^{\text{insp}}/\beta$ on $\Lambda_{i}^{\prime }$ (increase of $\Lambda_{i}^{\prime }$ with an increase of ${\text{Re}}_{1}^{\text{insp}}/\beta$). Generally speaking, these results show that the overall efficiency of water extraction from the lungs is not very sensitive to ${\text{Re}}_{1}^{\text{insp}}/\beta$ and therefore to the size of the person and to the intensity of a possible effort. Moreover, as mentioned previously, in the simplified framework, the dimensionless model is not dependent on the atmospheric conditions, and so does the overall efficiency of water extraction. Consequently, we can conclude that, whatever the breathing conditions (effort or rest, size of the person, atmospheric conditions), approximately 33% of the water evaporated during inspiration is actually condensed in the lungs during expiration, as a consequence of the cooled mucosa. It is a significant proportion, limiting the hydric and heat losses markedly. The plots of η_*i*_max__ as a function of ${\text{Re}}_{1}^{\text{insp}}/\beta$ are commented below.

In order to analyze how the water extraction by the ventilation is distributed in the lungs, we show, in Figure 7D, the ratio of the amount of water extracted per unit of time from the mucosa in generation *i*, *W*_*i*_, to the total amount of water extracted per unit of time from the lungs by the ventilation, *W*, as a function of *i*, for different values of ${\text{Re}}_{1}^{\text{insp}}/\beta$ (going from 40, corresponding to a newborn at rest, to 1,000, corresponding to an adult exercising). ϕ/ψ = 1 is used but, actually, these profiles are quite insensitive to ϕ/ψ (within the range of its possible values, approximately 0.3–1). Interestingly, the results show that, for ${\text{Re}}_{1}^{\text{insp}}/\beta$ below a certain threshold, it is in the trachea that the maximal water loss occurs while, for larger values of ${\text{Re}}_{1}^{\text{insp}}/\beta$, the generation in which the maximal water loss occurs is gradually further and further down in the bronchial tree. A similar behavior has been observed in a previous work about the allometric analysis of heat dissipation in mammal lungs (Sobac et al., 2020). It can be understood if we rewrite Equation (14) as:

(32) $$\begin{array}{l} {\tilde{C}}_{i}^{\text{insp}}-{\tilde{C}}_{i-1}^{\text{insp}}=\left({\text{Ψ}}_{i}^{\text{insp}}-1\right)\left({\tilde{C}}_{\mu ,i}-{\tilde{C}}_{i}^{\text{insp}}\right) \end{array}$$

This equation reveals that the amount of water evaporated in generation *i* during inspiration is proportional to the product of a driving force, ${\tilde{C}}_{\mu ,i}-{\tilde{C}}_{i}^{\text{insp}}$, tending to zero as *i* increases, and the coefficient ${\text{Ψ}}_{i}^{\text{insp}}-1$, characterizing the ability of the transport phenomena within the lumen of an airway in generation *i* to condition the air, increasing with *i* but tending to 0 as ${\text{Re}}_{1}^{\text{insp}}/\beta$ increases (see Equation 21). As a consequence, there is necessarily a threshold value of ${\text{Re}}_{1}^{\text{insp}}/\beta$ such that, when ${\text{Re}}_{1}^{\text{insp}}/\beta$ is larger than this threshold, the index of the generation in which the maximal water loss occurs, *i*_max_, is larger than 1.

Actually, this progression in the lungs of the location where the maximum of water is extracted makes it possible to highlight a subtle interaction between phenomena, contributing to the fact that the overall efficiency of water extraction from the lungs, η, is not very sensitive to the breathing conditions (see Figures 7B,C). Indeed, this efficiency is logically strongly linked to the processes taking place in the generation in which the maximum amount of water is extracted and, more precisely, to the value of $\Lambda_{i}^{\prime }$ in this generation. It is especially highlighted in Figures 7B,C, where we see that η, the overall efficiency of water extraction, and η_*i*_max__, the efficiency of water extraction in the generation in which the maximal amount of water is extracted, are quite close to each other. Interestingly, if we look combinedly at Figures 7B,D (both generated with ϕ/ψ = 1), we see that the value of $\Lambda_{i}^{\prime }$ for *i* = *i*_max_ is almost independent of ${\text{Re}}_{1}^{\text{insp}}/\beta$: it is equal to 0.264 for ${\text{Re}}_{1}^{\text{insp}}/\beta =40$ (*i*_max_ = 1), to 0.265 for ${\text{Re}}_{1}^{\text{insp}}/\beta =260$ (*i*_max_ = 5) and to 0.263 for ${\text{Re}}_{1}^{\text{insp}}/\beta =1,000$ (*i*_max_ = 8). In other words, even if $\Lambda_{i}^{\prime }$ can vary strongly depending on the values of ${\text{Re}}_{1}^{\text{insp}}/\beta$ and *i* (see Figure 6B), its value in the generation in which the maximal amount of water is extracted is almost constant, due to the progression of this generation down the bronchial tree. It explains why η_*i*_max__ is almost independent of ${\text{Re}}_{1}^{\text{insp}}/\beta$ in Figure 7B (also generated for ϕ/ψ = 1). To complete this analysis, it is worth to mention that the sudden changes observed in the plot of η_*i*_max__ in Figures 7B,C are due to the successive increases of the index of the generation in which the maximal amount of water is extracted. It is clearly observed when comparing Figure 7B and Figure 7E (both generated for ϕ/ψ = 1): the sudden changes of η_*i*_max__ and *i*_max_ are observed for the same values of ${\text{Re}}_{1}^{\text{insp}}/\beta$.

In Figure 7E, the index of the generation in which the maximal amount of water is extracted from the mucosa, *i*_max_, is plotted as a function of ${\text{Re}}_{1}^{\text{insp}}/\beta$ (for ϕ/ψ = 1). Coherently with the results presented in Figure 7D, we see that *i*_max_ = 1 for ${\text{Re}}_{1}^{\text{insp}}/\beta$ below a certain threshold (around 60) and that *i*_max_ is an increasing function of ${\text{Re}}_{1}^{\text{insp}}/\beta$ when the latter is larger than this threshold. If we consider that the mucosa is at body temperature (i.e., that ${\tilde{C}}_{\mu ,i}=1$) and that Sh ∝ (Re/β)^1/2^, the equations of the model can be solved analytically yielding:

(33) $$\begin{array}{l} i_{\text{max}}=\max\left(1,a+\frac{\log\left(\frac{{\text{Re}}_{1}^{\text{insp}}}{\beta }\right)}{\log\left(2h\right)}\right) \end{array}$$

with *a* a constant and max(*x, y*) the maximum of the values of *x* and *y*. This relation appears to generalize the one derived in Sobac et al. (2020), only relating *i*_max_ to the mass of the body, at rest. This scaling law is plotted in Figure 7E, with *a* chosen such that *i*_max_ = 2 for ${\text{Re}}_{1}^{\text{insp}}/\beta =50$. We see that it allows a good estimation of the increase of *i*_max_ with ${\text{Re}}_{1}^{\text{insp}}/\beta$.

Finally, the time average of the evaporation rate in the trachea, *E*_1_, is plotted as a function the body mass in Figure 7F. Two situations are considered: a rest situation (ϕ = ψ = 1) and an effort (ϕ = 2 and ψ = 6). *T*_0_ = 33°C and RH_0_ = 0.9 are used, as these are reference values for a human adult breathing through the nose in mild environmental conditions (Mcfadden et al., 1985; Elad et al., 2008). These values vary during an effort or between a child and an adult, but keeping them constant allows analyzing the sole influence of the lungs on the water exchanges within them. We report here *E*_1_ since, for all the considered situations, it is in the trachea that the highest evaporation rate is calculated. We see that *E*_1_ logically increases when an effort is realized and that it decreases if *M* increases. This decrease with the mass is a consequence of the increase of ${\text{Re}}_{1}^{\text{insp}}/\beta$ with *M*. Consequently, ${\text{Ψ}}_{i}^{\text{insp}}$, characterizing the ability of the transport phenomena within the lumen to condition the air, decreases with an increase of *M*, and so does *E*_1_.

### 3.2. Adult in Various Situations

**Figure 8:**
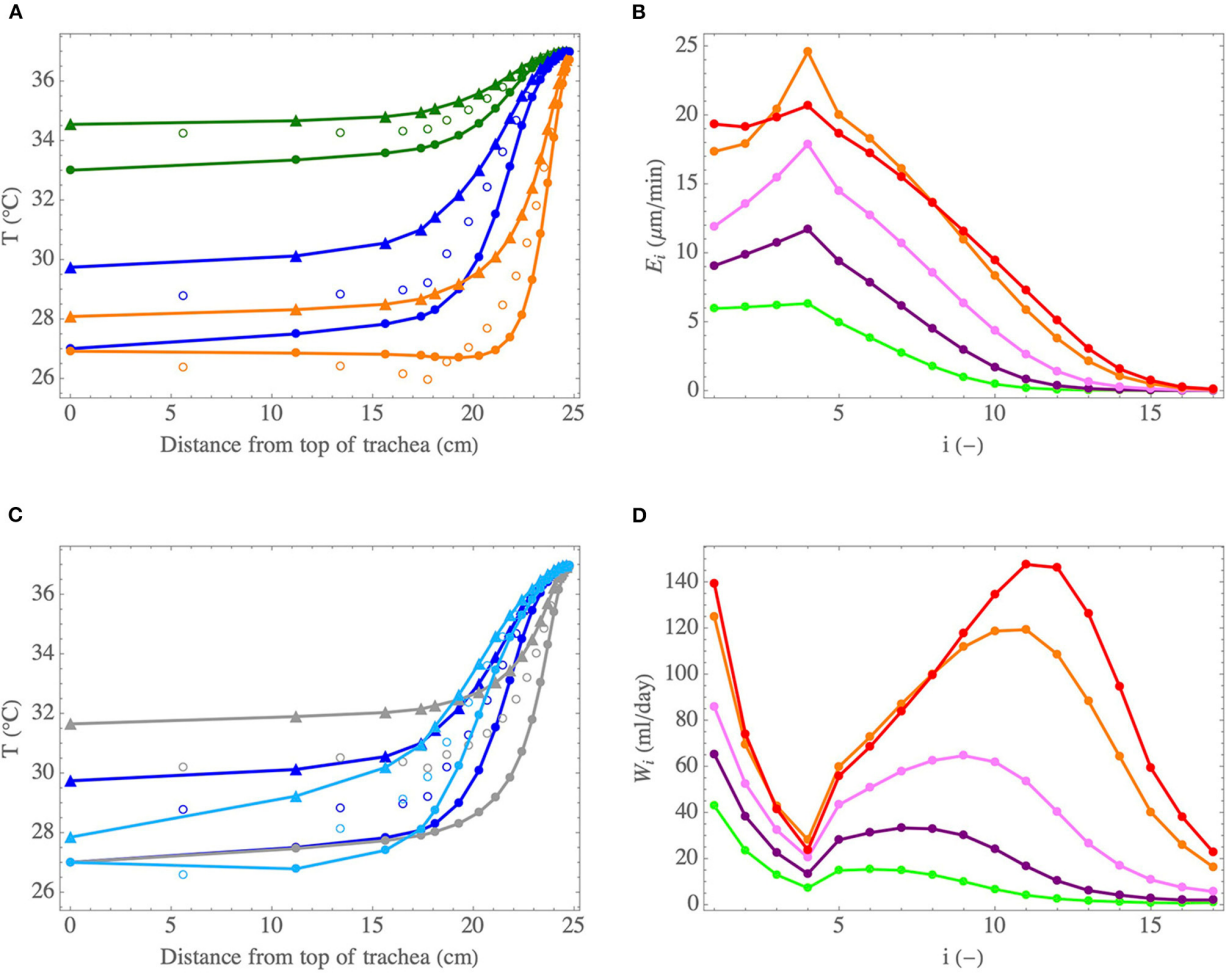
**(A)** Temperature profiles in the lumen of the lungs, during inspiration (full circles) and expiration (triangles), for cases I (•) (at rest and breathing by the nose in mild environmental conditions), II (•) (intubated in mild environmental conditions or breathing at rest very cold air by the nose) and VIII (•) (intense exercise and breathing by the mouth in mild environmental conditions). The empty circles indicate the calculated values of *T*_μ_. **(B)** Evaporation rate in an airway in generation *i*, *E*_*i*_, as a function of *i*, when breathing by the mouth during an exercise in mild environmental conditions, for different inspiration flow rates: 15 l/min (•), 30 l/min (•), 60 l/min (•), and 120 l/min (•). Case IX (•) is also for an inspiration flow rate of 120 l/min, but when breathing a cold and dry air. **(C)** Temperature profiles in the lumen of the lungs, during inspiration (full circles) and expiration (triangles), for cases II (•) (normal pressure), III (•) (decreased pressure) and IV (•) (increased pressure). The empty circles indicate the calculated values of *T*_μ_. **(D)** Total amount of water evaporated per unit of time in generation *i*, *W*_*i*_, as a function of *i*, when breathing by the mouth during an exercise and for different inspiration flow rates. Same color codes than for **(B)**.

In this section, we analyze the heat and water exchanges in the lungs of a human adult in various situations (varying environmental conditions, varying rate of exercising…), using the complete model (see section 2.2.1), with γ = 1. Two sets of results, presenting key quantities related to these exchanges, are reported in Tables 3, 4 and Figure 8. These results are generated using values of *L*_*i*_, *R*_*i*_, and *n* derived from morphometric data, scaled to yield a functional residual capacity of 3 l (Karamaoun et al., 2016). These values are given in Table 2.

**Table 2:** Morphometric data, scaled to yield a functional residual capacity of 3 l (Karamaoun et al., 2016).

***i***	***L*_*i*_ (cm)**	***R*_*i*_ (cm)**	**β_*i*_ (-)**	**${\text{Re}}_{i}^{\text{insp}}$ (-)**
1	11.19	0.71	16	1,315
2	4.44	0.48	9	971
3	1.77	0.33	5	717
4	0.71	0.22	3	524
5	1.17	0.18	7	333
6	0.99	0.14	7	210
7	0.84	0.11	8	132
8	0.71	0.09	8	82
9	0.60	0.07	8	50
10	0.50	0.06	8	30
11	0.43	0.05	8	18
12	0.35	0.04	8	10
13	0.30	0.04	8	6
14	0.24	0.03	7	3
15	0.19	0.03	6	2
16	0.19	0.03	7	1
17	0.15	0.02	6	1

*The values of ${\text{Re}}_{i}^{\text{insp}}$ are calculated using Equation (13), with ν = 1.7 ×10^−5^ m^2^ s^−1^ and Q^insp^ = 15 l min^−1^*.

**Table 3:** Calculation results, breathing by the nose or intubated, in mild or very cold air, at normal, low or high ambient pressure.

	**Case**	***T*_0_ (°C)**	**RH_**0**_ (-)**	**P (J/s)**	**W (l/day)**	**$\bar{\eta }$ (-)**	**η (-)**	***E*_max_ (μm/min)**
I (•)	Mild air	33	0.9	2.2	0.07	0.53	0.56	2.4
II (•)	Intubated / Cold - normal *p*_*atm*_	27	0.4	6.3	0.21	0.55	0.58	8.1
III (•)	Cold - low *p*_*atm*_	27	0.4	5.1	0.18	0.45	0.50	9.4
IV (•)	Cold - high *p*_*atm*_	27	0.4	7.5	0.24	0.65	0.68	5.9

*All the results are generated with Q^insp^ = 15 l min^−1^ and t_w_ = 2, 000 s. E_max_ is the highest value of the time averaged evaporation rate, which is calculated in the fourth generation for all the calculations, except for the one at decreased pressure, where it is in the first generation that the maximal evaporation rate is calculated*.

**Table 4:** Calculation results, breathing by the mouth during an exercise.

	**Case**	***Q*^insp^ (l/min)**	***t*_*w*_ (s)**	***P* (J/s)**	***W* (l/day)**	**$\bar{\eta }$ (-)**	**η (-)**	***E*_max_ (μm/min)**
V (•)	At rest	15	2,000	6.6	0.22	0.58	0.61	6.3
VI (•)	Exercise	30	1,000	12.9	0.43	0.56	0.60	11.7
VII (•)	Exercise	60	900	23.1	0.79	0.50	0.55	17.9
VIII (•)	Exercise	120	800	41.2	1.44	0.45	0.50	24.6
IX (•)	Exercise - cold	120	800	66.0	1.67	0.46	0.44	20.7

*All the results are obtained using an ambient air at 27°C and at a relative humidity of 40%, except for case •, where an ambient air at 5°C and at a relative humidity of 1% is used. E_max_ is the highest value of the time averaged evaporation rate, which is calculated in the fourth generation for all the calculations*.

The results presented in Table 3 are computed for a person at rest, breathing with *Q*^insp^ = 15 l min^−1^, for various conditions at the top of the trachea during inspiration (i.e., various values of *T*_0_, RH_0_ and of the atmospheric pressure). *t*_*w*_ = 2, 000 s is used. There is a significant uncertainty on the value of this parameter. However, we have observed that the model is not very sensitive to its value (it is the square root of *t*_*w*_ that appears in the dimensionless number Λ, see Equation 8). Case I (•) is an adult, with *T*_0_ = 33°C and RH_0_ = 0.9. These values were measured experimentally for a human adult during a rest inspiration by the nose in a room with an ambient temperature of 27°C and a relative humidity of 40% (Mcfadden et al., 1985). These environmental conditions are hereafter referred to as “mild environmental conditions”. Case II (•) is an adult, still breathing at rest in a room with an air at 27°C and at a relative humidity of 40%, but being intubated (for example during anesthesia) or having had a tracheostomy. In this case, we can consider that *T*_0_ = 27°C and RH_0_ = 0.4, as the upper tract is bypassed. According to the results of Mcfadden et al. (1985), these values of *T*_0_ and RH_0_ are also representative of those that one would have at the top of the trachea while inspiring at rest and by the nose an air with a temperature below 0°C. Finally, cases III (•) and IV (•) use the same values of *T*_0_ and RH_0_ as case II (•) (i.e., breathing in very cold and dry air), but correspond to a hypo or a hyperbaric situation. Case III (•) is for an ambient air pressure of 0.3 bar (for instance at the top of a very high mountain) and case IV (•) is for an ambient air pressure of 10 bar (for instance when diving at a depth of 100 m). In order to take into account the influence of the pressure, the kinetic theory of gases is used to express the pressure dependence of physicochemical parameters involved in the model. Accordingly, the Reynolds numbers of the flow in an airway (during inspiration and expiration) appear to be proportional to the pressure. Consequently, the Sherwood and Nusselt numbers are also increasing function of the pressure. However, as Sh and Nu increase slower than Re (see Figure 3), the dimensionless numbers Ψ and $\bar{\text{Ψ}}$ (see Equations 3 and 6) decrease if the pressure increases. Moreover, since the diffusion coefficient of water in air is inversely proportional to the pressure, we see that Λ and Φ increase with the pressure. In conclusion, an increase of the pressure leads to a decreased ability of the transport phenomena in the lumen to condition the air (decrease of Ψ and $\bar{\text{Ψ}}$), to an increased part of the energy extracted to heat the air (increase of Φ) and helps to maintain the mucosa at the body temperature (increase of Λ).

The results presented in Table 4 are generated for various values of the inspiration flow rate, to analyze the impact of exercising on the heat and water exchanges in the lungs. Breathing by the mouth is considered. To take into account the conditioning of the air by the pharynx and the larynx, we add two single airways to the geometrical model of the lungs presented in Table 2 (one airway in generation −1, corresponding to the pharynx, with a length of 2 cm and a radius of 1 cm, and one airway in generation 0, corresponding to the larynx, with a length of 3 cm and a radius of 1 cm, see Daviskas et al., 1990). We consider that, during inspiration, the atmospheric conditions can be applied at the top of the pharynx. Except for case •, the temperature and the relative humidity of the ambient are taken equal to 27°C and 40%, respectively. Case V (•) is a reference case, with an inspiration flow rate of 15 l min^−1^, corresponding to a rest situation. Accordingly, *t*_*w*_ is set to 2,000 s. Then, three situations of exercise with an increasing inspiration flow rate are considered: cases VI (•) (inspiration flow rate of 30 l min^−1^), VII (•) (inspiration flow rate of 60 l min^−1^) and VIII (•) (inspiration flow rate of 120 l min^−1^). To take into account the increase of the cardiac flow rate with the effort intensity, *t*_*w*_ is progressively decreased. To give an idea, if we assume that *t*_*w*_ = 2, 000 s corresponds to a heart rate of 60 bpm, *t*_*w*_ = 1, 000 s would correspond to a heart rate of 120 bpm, *t*_*w*_ = 900 s to a heart rate of 133 bpm, and *t*_*w*_ = 800 s to a heart rate of 150 bpm. Finally, case IX (•) corresponds to case VIII (•), except that an ambient air with a temperature of 5°C and a relative humidity of 1% is considered, everything else being constant. This represents a situation where the sportsman is outside and breathes a cold and dry air.

The analysis of the results presented in Tables 3, 4 and Figure 8 allows highlighting several interesting points. First, globally and coherently with the results presented in section 3.1, we observe in Figures 8A,C that the air is at body temperature before reaching the end of the bronchial region at inspiration and that a significant cooling of the air occurs along the tract during expiration, in all the conditions represented on these figures.

Case I (•) considers the reference situation of an adult breathing at rest and by the nose in mild environmental conditions. First, it is worth mentioning that the results presented in Figure 8A and in Table 3 for this case are in good agreement with the experimental data of Mcfadden et al. (1985), as well as with the numerical simulations of Warren et al. (2010) and Wu et al. (2014). Notably, our model predicts a cooling of the air during expiration yielding a temperature difference at the top of the trachea, between inspiration and expiration, of approximately 1.5°C, very close to the one measured by Mcfadden et al. (1985) and the one calculated by Wu et al. (2014), in the same conditions. The latter performed CFD simulations considering turbulence and a full description of the bifurcations for several subjects whose lung geometries were obtained by CT-Scans. This good comparison supports our approach. A significant cooling of the air along the respiratory tract during expiration is calculated, due to the low values of Λ_*i*_ compared to one. The ability of the submucosal glands to replenish the ASL with water has been the subject of many research works and a maximal rate of replenishment close to 20–25 μmmin^−1^ is often put forward (see the review of Widdicombe, 2002). We see in Table 3 that, at rest and breathing by the nose in mild environmental conditions, the highest value of the evaporation rate (2.4 μmmin^−1^, calculated in the fourth generation) is one order of magnitude smaller than this replenishment rate. Consequently, the evaporation is unlikely to generate a dehydration of the ASL in the considered conditions. But, in the case of a disease associated with airway dysfunction, such as cystic fibrosis, where the operation of the submucosal glands is impaired (Joo et al., 2006), it is worth noting that ventilation could dehydrate the ASL quickly (in a few minutes, as the ASL is approximately 10 μm thick). At rest, around 10 generations (i.e., over a distance of approximately 23 cm from the top of the trachea, according to Table 2) are significantly involved in the air conditioning (see Figure 8A). It is interesting to note that it corresponds approximately to the generations with submucosal glands, as they are mainly present in airways with a diameter larger than 2 mm (Fahy and Burton, 2010). The values of *P* and *W* calculated for this reference case I (•) are 2.2 J s^−1^ and 70 ml day^−1^, respectively (see Table 3). These values are much smaller than the ones mentioned in the introduction for the maximal amounts of heat and water that could be extracted, per unit of time, from the body by the respiration (13 J s^−1^ and 360 ml day^−1^). Such a difference can easily be understood by the fact that we consider here (i) milder environmental conditions, (ii) the sole lungs rather than the entire respiratory tract, and (iii) an advanced description of the heat and water exchanges with a significant cooling occurring during expiration.

For case II (•) (intubated in mild environmental conditions or breathing at rest very cold air by the nose) and case VIII (•) (intense exercising in mild environmental conditions), we observe a large increase of the evaporation rate, when compared to case I (•) (see Tables 3, 4 and Figure 8B for the exercise case). It is due to the enhanced temperature and concentration gradients developing in the lungs in these situations. Also, more generations than for case I (•) (i.e., over a larger distance from the top of the trachea) are involved in the conditioning for these two cases (see Figure 8A). In generation 10, an evaporation rate close to 2 μmmin^−1^ is calculated for case II (•) (not shown in the tables/figures) and up to 10 μmmin^−1^ for case VIII (•) (see Figure 8B). Moreover, when exercising, the evaporation rate in the fourth generation becomes of the same order of magnitude than the maximal replenishment rate of the submucosal glands (see Figure 8B). In Figure 8A, we observe a cooling of the mucosa below 30°C in the five first generations for case II (•) (i.e., over a distance of approximately 19 cm from the top of the trachea) and in the 9 first generations for case VIII (•) (i.e., over a distance of approximately 22 centimeters from the top of the trachea). For the latter, an evaporative cooling of the mucosa below the air temperature in the 5 first generations is even calculated, as well as an almost constant air temperature in the 7 first generations (i.e., over a distance of approximately 21 centimeters from the top of the trachea): the energy extracted from the mucosa to evaporate water is so high, due to the high value of the Sherwood numbers, that the mucosa becomes even colder than the lumen and, consequently, the air in the lumen is not heated. These results show why it is very important to condition the air during an intubation or to be very careful when exercising or breathing cold and dry air while suffering from a pathology associated with an airway dysfunction.

Coherently with what is mentioned above, we see in Table 3 and Figure 8C that increasing the pressure from 0.3 to 10 bar leads to (i) an increase of $\bar{\eta }$ and η (due to the increasing values of Λ_*i*_, that becomes larger than one in the proximal generations for an ambient pressure of 10 bar), (ii) a decrease of the evaporation rate and hence to the mobilization of an increasing number of generations for the air conditioning (due to the decrease of ${\text{Ψ}}_{i}^{\text{insp}}$ and the increase of Φ_*i*_), (iii) an increase of the total amount of water/energy extracted by the ventilation, as a consequence of the increase of the efficiencies.

Exercising leads obviously to an increase of the total amount of heat/water extracted from the lungs by the ventilation. Table 4 shows that, when exercising and breathing by the mouth, *P* and *W* increase by approximately a factor 6 when *Q*^insp^ is increased from 15 l min^−1^ to 120 l min^−1^, in the same atmospheric conditions. The values of *P* calculated for *Q*^insp^ = 120 l min^−1^ remain limited when compared to the amount of heat that has to be dissipated by the body during an effort (which can reach 1,000 J s^−1^). On the other hand, regarding *W*, we see in Table 4 that it can reach quite high values when exercising, showing that the evaporation of water in the lungs may contribute significantly to dehydration during a long and intense effort. Whatever the ventilation rate, it is in generation 4 that the maximal evaporation rate, *E*_max_, is calculated (see Figure 8B). It is due to the small aspect ratio of the airways in this generation (see Table 2). Consequently, the flow in these airways is largely undeveloped, leading to high concentration gradients at the ASL–lumen interface. An increase of the ventilation rate logically implies an increase of *E*_max_, as well as of the number of generations mobilized to condition the air (due to the decrease of Ψ and $\bar{\text{Ψ}}$). At high ventilation rates, the calculated values of *E*_*i*_ indicate a possible problem in the proximal generations regarding the hydration of the ASL, as they are of the same order of magnitude than the maximal rate of replenishment mentioned above (see Figure 8B). Moreover, beyond generation 10, where there are no submucosal glands, the evaporation rates calculated at high ventilation rates becomes significant (as high as the ones calculated in the proximal generations at rest). It could disrupt the mucus balance in these generations. Overall, the results show that there is likely an intensity threshold in the exercise above which evaporation in the lungs leads to dehydration of the ASL, given that it can no longer be compensated by the submucosal glands. This threshold depends of course on the atmospheric conditions. It is interesting to note that similar results were previously obtained by Combes et al. (2019) and Karamaoun et al. (2019). These authors highlighted a deterioration of the epithelium of the bronchial region of the lungs above a ventilation rate of about 80 l min^−1^ when exercising continuously, due to the dehydration of the mucus.

We observe two maxima in the plot of *W*_*i*_ as a function of *i* (see Figure 8D). The first one is in the trachea (due to its large exchange surface and the high concentration gradients met there), the other one in a more distal generation, further and further in the lungs when the ventilation rate is increased. This is coherent with the results presented in Figure 7D.

The comparison of cases IX (•) (exercising at high ventilation rate in cold air) and VIII (•) (exercising at high ventilation rate in mild air) shows that, when an athlete breathes cold and dry air, the amounts of heat and water extracted from his lungs by ventilation increase, compared to a mild environment (see Table 4). It is logical, the lower the temperature of the inspired air, the higher the amounts of heat and water needed to bring it to body temperature and to water saturation. Nevertheless, it can be seen that the amount of heat extracted in case IX (•) is significantly larger (60%) than the one extracted in case VIII (•), while the amount of water extracted in case IX (•) is only 15% larger than the one extracted in case VIII (•). This is because the saturation concentration of water in air depends on the temperature in a non-linear way. We can calculate that the sensible heat needed to condition the air in case IX (•) is more than 300% larger than the one needed to condition the air in case VIII (•), but that the amount of water needed (and the corresponding latent heat) is only 30% larger. In addition, we can see in Figures 8B,D that, in the first generations, the evaporation rates encountered are almost identical for both cases. This is because two opposite effects offset each other. Inhaling cold air tends to increase the difference in water concentration between the mucosa (if it remains at the same temperature) and the core of the airway lumen, which is in favor of an increase in the evaporation rate. However, the inspiration of this cold air results in a significant cooling of the mucosa, because the vascularization cannot maintain it at body temperature. This results in a decrease in the difference in water concentration between the mucosa and the core of the lumen, which tends to decrease the evaporation rate. We see in Figure 8D that it is actually in distal generations that the difference between the two cases is marked, with more water removed from the lungs in the case where cold air is inhaled.

Finally, regarding the overall efficiencies of heat and water extraction from the lungs ($\bar{\eta }$ and η), we see that, as in the simplified framework, they do not vary a lot with the breathing conditions, except when the pressure is varied (see Tables 3, 4). At atmospheric pressure, whatever the situation, there is a significant cooling and condensation at expiration: around 45% of the heat and water used to condition the air during inspiration are transferred back to the mucosa during expiration. Nonetheless, we may note that these efficiencies decrease slightly with the ventilation rate (see Table 4); this is coherent with the results in Figure 7C. The efficiencies calculated in this section are slightly smaller than within the simplified framework; this is due to the change of geometrical model. Generally speaking, the results show that the exchanges of heat and water between the lungs and the environment involve evaporation and condensation phenomena, both of significant importance. These exchanges increase in amplitude when exercising or when breathing a cold and dry air.

## 4. Conclusion

In this paper, a new theoretical model allowing a comprehensive analysis of the heat and water exchanges in the human lungs (from a newborn to an adult) is developed. It is based on their local description in the lumen of the airways and in the surrounding tissues. It is easy to tune to any geometry. The model is presented in a general and dimensionless framework with explicitly stated assumptions and a strong physiological background. The main originality of this model is its description of the local phenomena in a hierarchical structure based on well-defined and well-established dimensionless numbers. This provides a certain rigor and generality to the model and its results, which can hardly be reached by more classical approaches based on directly solving the conservation laws in a complex domain.

As summarized in section 2.2.3 (see notably Figure 5 and Table 1), the key dimensionless numbers controlling the heat and water exchanges in the lungs are: Ψ and $\bar{\text{Ψ}}$, characterizing the ability of the transport phenomena within the lumen to condition the air, and Λ, characterizing the competition between vascularization and heat withdrawal at the level of the tissues surrounding an airway. These dimensionless numbers were thoroughly analyzed and scaling laws were derived. A simplified version of the model, independent of the atmospheric conditions in its dimensionless form, has been developed. In this simplified framework, the ratio of the Reynolds number of the flow in the trachea to its aspect ratio (Re/β) has been found to be the key variable parameter.

### 4.1. Summary of the Main Physiological Findings

The model allows giving new insights into the heat and water exchanges in the lungs. Several interesting elements have been highlighted.

The bronchial region of the lungs is able to condition the air in all the situations considered in this work even if, sometimes, for instance when exercising or at high pressure, distal generations have to be involved (beyond generation 10). Based on the values of the dimensionless parameter Ψ, it has been shown that these distal generations are super-conditioners. In these generations, the regime of water and heat transfer between the ASL and the lumen is the transition one (Sherwood and Nusselt numbers almost constant), with Ψ increasing faster than exponentially with the generation index, while, in the proximal generations, the regime of water and heat transfer between the ASL and the lumen is the boundary layer one (Sherwood and Nusselt numbers proportional to the square root of the Reynolds number). Additionally, the model predicts that newborn airways are better conditioners than adult ones. It also shows that the heat used to evaporate water accounts for more than 80% of the heat extracted from the lungs, except in particular situations (breathing a very cold air or being at high pressure). In parallel, the results quantify the key role of the submucosal glands. Their presence in the first 10 generations of the lungs of an adult is an important feature of the human evolution to allow, by keeping the mucus wet despite of the evaporation of water, the first generations of the lungs to exercise their function of mucociliary clearance.

Due to the small value of Λ compared to 1, the vascularization of the tissues surrounding the airways is not able to maintain these tissues at body temperature during inspiration. Consequently, during expiration, a significant cooling of the air and condensation of water occur. Due to a subtle interaction between different phenomena, it appears that, at a given pressure and for a fixed geometrical representation of the lungs, the overall efficiencies of heat and water removal from the lungs are almost independent of the breathing conditions (effort or rest, size of the person, atmospheric conditions) (see section 3.1). For instance, when the simplified framework is used, we calculate that approximately 33% of the heat and water used to condition the air during inspiration are transferred back to the mucosa during expiration (45% when using the morphometric data given in Table 2).

Except for the trachea (when the morphometric data given in Table 2 are used), the index of the generation in which the maximal amount of water is extracted (and also the maximal amount of heat) is an increasing function of the Reynolds number in the trachea, divided by its aspect ratio. Accordingly, it is also an increasing function of the body mass. Consequently, the generation in which the maximal amount of water is extracted is further and further down in the bronchial tree during the life of a human (trachea excepted). It also progresses down the tree with the intensity of an effort; for an adult, it can be beyond generation 10 during an intense exercice.

In acute situations, such as suffering from a pathology with airway dysfunction, when being intubated, when exercising above a critical threshold, the heat and water exchanges in the lungs may be critical regarding mucus hydration and exposure to cold of the mucosa. In proximal generations, the evaporation may overwhelm the ability of the submucosal glands to replenish the ASL with water, and it can even be significant in distal generations, where there are no submucosal glands. During an intense and long exercise, the evaporation in the lungs can contribute to dehydration of the body. In some situations, the cooling of the mucosa, mainly induced by the evaporation, may also be very important; the mucosa can even reach temperature colder than the inspired air. Finally, the results show that breathing cold air can significantly increase the exchanges between the lungs and the environment, which can be critical regarding disease transmission.

### 4.2. Model Limitations and Future Work

Despite being quite general, the model has several limitations. First, it is well-known that the flow in the trachea and in the few generations downstream can be turbulent, especially at high inspiration and expiration flow rates. This feature is not included in the model as only steady states are considered, but it has limited impact on the Sherwood and Nusselt numbers. Indeed, in the proximal generations of the lungs, the flow-establishment length is usually significantly larger than the length of the airways, and the undeveloped character of the flow in these proximal airways has more impact on the transfers than the potentially turbulent nature of the flow (Pedley et al., 1970a,b). As mentioned previously, the CFD simulations of the momentum heat and mass transport equations in a single airway, used to construct the correlations of Sh and Nu, account for this undeveloped character of the flow by imposing a constant axial velocity at the inlet of an airway (Supplementary Equation 9 in the Supplementary Material). Another limitation is the simplified way to treat the bifurcations (by imposing constant velocity, concentration and temperature at the inlet of an airway, see Supplementary Equations 8, 9 in the Supplementary Material). The model is also based on a representation of the lungs in which all airways are right circular cylinders. It is an approximation in the first few generations (except for the trachea), in which the airways have a bended shape.

Two additional limitations of the model are related to the collection of data needed to use it and to validate it. Applying the model to a specific person could be quite relevant in some critical situations, for instance to finely tune an air humidification system during the anesthesia of someone with airway dysfunction. However, it would require the collection of a large amount of data relating to the geometry of the lungs of this person, as these are very person-dependent. In addition, as already mentioned previously, there is little experimental data to confront the model with, in order to fully validate/refine it. Regarding the geometrical representation of the lungs, computed tomography or conventional magnetic resonance are imaging technologies that can provide valuable information. Their resolution limits the clear visualization of airways located from the ninth generation on (Lewis et al., 2005; Belchi et al., 2018), but it not an issue regarding heat and mass transfers as it is in the proximal generations that they mainly take place (except at high ventilation rate). With respect to the validation of the model, some data could also be collected thanks to advances in imaging methods. Specifically, thermal imaging of breathing could potentially provide dynamic thermal data during inspiration and expiration, for the upper respiratory tract (see for instance Duong et al., 2012, 2017).

## Data Availability Statement

The original contributions presented in the study are included in the article/Supplementary Material, further inquiries can be directed to the corresponding author/s.

## Author Contributions

AB, BH, and BS: conceptualization, methodology, model, and code development. BH, AN, JR, CR, and BS: investigation and writing—review and editing. BH: funding acquisition and writing—original draft preparation. BH and BS: supervision. All authors have read and agreed to the published version of the manuscript.

## Conflict of Interest

The authors declare that the research was conducted in the absence of any commercial or financial relationships that could be construed as a potential conflict of interest.

## Figures and Tables

**Table:** 

*Roman symbols*	
*C*	Molar concentration of water in air, molm^−3^
*c*_*p*_	Heat capacity at constant pressure, Jkg^−1^ K^−1^
*D*	Diffusion coefficient of water in air, m^2^ s^−1^
*E*	Evaporation flux of the water contained in the ASL, m s^−1^
*f*	Correlation to express the Sherwood number as a function of Re and β, -
*g*	Correlation to express the Nusselt number as a function of Re and β, -
*h*	Ratio of the radii of airways in two successive generations, -
*k*	Mass transfer coefficient between the mucosa and the airway, m s^−1^
*L*	Length of the airway, m
$L_{m}$	Molar latent heat of vaporization, J mol^−1^
*M*	Mass of the body, kg
M	Molar mass of water, kg mol^−1^
*n*	Number of generations in the bronchial region of the lungs, -
Nu	Nusselt number of the heat transfer between the mucosa and the lumen, -
*P*	Power extracted from a tissue, J s^−1^
Pr	Prandtl number of the air, -
*Q*	Volumetric flow rate of air, m^3^ s^−1^
*R*	Radius of the airway, m
Re	Reynolds number of the flow in the airway, -
RH	Relative humidity of the air, -
Sc	Schmidt number of the air, -
Sh	Sherwood number of the mass transfer between the mucosa and the lumen, -
*t*	Time, s
*T*	Temperature, K
*u*	Heat transfer coefficient between the mucosa and the airway, W m^−2^ K^−1^
*v*	Velocity of the air, m s^−1^
*W*	Rate of water removal from the ASL, m^3^ s^−1^
	
*Greek symbols*	
α	Thermal diffusivity, m^2^ s^−1^
β	Aspect ratio of the airway, -
δ	Thickness, m
η	Efficiency of the water extraction from the mucosa, -
$\bar{\eta }$	Efficiency of the heat extraction from the bronchial wall, -
γ	Ratio of the expiration duration to the inspiration duration, -
λ	Thermal conductivity, W m^−1^ K^−1^
Λ	Dimensionless number, see Equation (8)
Λ′	Dimensionless number, see Equation (23)
ν	Kinematic viscosity, m^2^ s^−1^
ϕ	Factor accounting for the increase of the cardiac flow rate during an effort, -
Φ	Dimensionless number, see Equation (9)
Ψ	Dimensionless number, see Equation (3)
$\bar{\text{Ψ}}$	Dimensionless number, see Equation (6)
ρ	Density, kg m^−3^
Θ	Dimensionless constant, -
ψ	Factor accounting for the increase of the ventilation rate during an effort, -
*Abbreviations, subscripts, and superscripts*	
-	No subscript in a physicochemical property refers to the air
ỹ	Dimensionless version of *y*
0	At the top of the trachea during inspiration
ASL	Airway Surface Liquid
av	Averaged on a flow cross-section
*b*	Body
exp	Related to the expiration
*l*	Liquid phase
*i*	In generation *i*
in	At the inlet of the airway
insp	Related to the inspiration
max	Maximal
sat	Saturated with water
SM	Supplementary Material
tot	Total respiratory cycle
out	At the outlet of the airway
*w*	Tissues composing the wall of the airway
μ	At the ASL–lumen interface
	
*Other symbols*	
[]	Velocity average on the entire lumen of the airway
