# Supplementary material for: Comprehensive Analysis of Heat and Water Exchanges in the Human Lungs
Source: Front Physiol. 2021 Jun 8;12:649497. doi: 10.3389/fphys.2021.649497 (PMC8217871; doi:10.3389/fphys.2021.649497)
Supplement: Supplementary file 1 [file Data_Sheet_1.PDF]

Heat and Water Exchanges in the Human Lungs - Supplementary Material

1 MAXIMAL AMOUNTS OF WATER AND HEAT EXTRACTED FROM THE LUNGS BY VENTILATION

The maximal amounts of heat and water that could be extracted per unit of time from the body by the respiration can be calculated easily as they correspond to a hypothetical situation in which the expired air would be saturated with water and at the body temperature. Let us consider a human, inspiring and expiring F times per unit of time a volume V^{insp} of ambient air at a temperature T_{amb} and a relative humidity RH_{amb}. The maximal amount of water, W_{max}, and the maximal amount of heat, P_{max}, that could be extracted per unit of time from the body by this respiration process are respectively:

$$W_{\text{max}} = F\,V^{\text{insp}}\left(C_{\text{sat}}(T_b) - \text{RH}_{\text{amb}}C_{\text{sat}}(T_{\text{amb}})\right)\frac{\mathcal{M}}{\rho_l} \tag{S1}$$

$$P_{\text{max}} = F\,V^{\text{insp}}\left(\rho c_p(T_b - T_{\text{amb}}) + \mathcal{L}_m\left(C_{\text{sat}}(T_b) - \text{RH}_{\text{amb}}C_{\text{sat}}(T_{\text{amb}})\right)\right) \tag{S2}$$

with $C_{\text{sat}}(T)$ the saturation concentration of water in air at the temperature T, T_b the body temperature, $\mathcal{M}$ the molar mass of water, ρ_l the density of liquid water (equal to ρ_w according to our assumptions, see section below), ρ the density of the air and c_p the heat capacity at constant pressure of the air.

The first equation S1 expresses that the maximal amount of water (in equivalent volume of liquid water) that could be extracted from the body by the ventilation per unit of time is equal to the product of $C_{\text{sat}}(T_b) - \text{RH}_{\text{amb}}C_{\text{sat}}(T_{\text{amb}})$, the number of moles that must be added to a unit of volume of air during its journey in the respiratory system to saturate it with water at the body temperature, of $\mathcal{M}/\rho_w$, a conversion factor to transform this number of moles into a volume of liquid water, of V^{insp}, the volume of air inspired and expired during each respiratory cycle, and of F, the number of respiratory cycles per unit of time.

Similarly, the second equation S2 expresses that the maximal amount of heat that could be extracted from the body by the ventilation per unit of time is equal to the amount of joules that must be supplied to a unit of volume of air during its journey in the respiratory system to fully condition it (i.e. to heat it up to body temperature and saturate it with water at this temperature), multiplied by $F\,V^{\text{insp}}$.

Let us now consider a human adult, inspiring and expiring $F = 15$ times per minute a volume $V^{\text{insp}} = 500$ ml of ambient air at a temperature $T_{\text{amb}} = 20$°C and a relative humidity $\text{RH}_{\text{amb}} = 0.6$. With the equations expressed above and using the values of the physicochemical properties provided in the next section, we estimate that $W_{\text{max}} \simeq 360$ ml/day and $P_{\text{max}} \simeq 13$ W.

Moreover, we can evaluate in equation S2 that $\rho c_p(T - T_{\text{amb}})$ is approximately 4 times smaller than $\mathcal{L}_m(C_{\text{sat}}(T_b) - \text{RH}_{\text{amb}}C_{\text{sat}}(T_{\text{amb}}))$, meaning that the amount of heat extracted from the body to heat the air is about four times smaller than the amount of heat extracted to evaporate water in the mucus in order to saturate the air. In other words, it is interesting to observe that the amount of heat extracted from the body to heat the air accounts for only 20% of the total amount of heat extracted, and that the large majority (80%) is actually used for water evaporation. Consequently, $P_{\text{max}} \simeq F\,V^{\text{insp}}\mathcal{L}_m\left(C_{\text{sat}}(T_b) - \text{RH}_{\text{amb}}C_{\text{sat}}(T_{\text{amb}})\right) \propto W_{\text{max}}$.

2 PHYSICOCHEMICAL PROPERTIES

As mentioned in the core of the paper, the physicochemical properties involved in the model are considered constant (i.e. independent of the local value of the temperature or the water concentration in the air). They are calculated using equations given in Sobac et al. (2015) and considering that the thermal properties of the ASL, the epithelium and the tissues surrounding the airways are those of liquid water (Warren et al., 2010). They are calculated at the body temperature, taken equal to 37°C, and, for the physicochemical properties of the air, at a relative humidity of 100% and at a pressure of 101325 Pa (except in two cases, detailed in the core of the paper).

The air is considered to be an ideal gas. Therefore, $C_{\text{sat}}(T) = p_{\text{sat}}(T)/(\mathcal{R}T)$, with $p_{\text{sat}}(T)$ the saturation pressure of water at the temperature T and $\mathcal{R}$ the ideal gas constant. $p_{\text{sat}}(T)$ is evaluated using the Clausius-Clapeyron equation, using the body temperature as the reference temperature.

The values of the physicochemical properties used in the model are given in the table below.

**Physicochemical property**	**Symbol**	**Value**	**Units**
Density of the air	ρ	1.138	kg m^{-3}
Density of the tissues composing the bronchial wall	ρ_w	993	kg m^{-3}
Diffusion coefficient of water in air	D	2.7×10^{-5}	$\text{m}^2\,\text{s}^{-1}$
Heat capacity at constant pressure of the air	c_p	1006	$\text{J kg}^{-1}\,\text{K}^{-1}$
Heat capacity at constant pressure of the tissues composing the bronchial wall	$c_{p,w}$	4180	$\text{J kg}^{-1}\,\text{K}^{-1}$
Ideal gas constant	$\mathcal{R}$	8.314	$\text{J mol}^{-1}\,\text{K}^{-1}$
Kinematic viscosity of the air	ν	1.7×10^{-5}	$\text{m}^2\,\text{s}^{-1}$
Molar latent heat of vaporization of water	$\mathcal{L}_m$	43470	J mol^{-1}
Molar mass of water	$\mathcal{M}$	18×10^{-3}	kg mol^{-1}
Prandtl number of the air	Pr	0.72	-
Saturation pressure of water in air at the body temperature	$p_{\text{sat}}(T_b)$	6283	$\text{kg m}^{-1}\,\text{s}^{-2}$
Schmidt number of the air	Sc	0.63	-
Temperature of the body	T_b	310.15	K
Thermal conductivity of the air	λ	0.027	$\text{W m}^{-1}\,\text{K}^{-1}$
Thermal conductivity of the tissues composing the bronchial wall	λ_w	0.62	$\text{W m}^{-1}\,\text{K}^{-1}$

3 HEAT AND WATER EXCHANGES IN A SINGLE AIRWAY

In this section of the supplementary material, the modeling and the numerical simulations at the scale of a single airway are fully detailed.

Heat and Water Transfers in the Lumen of the Airway

First, we consider the heat and water transfers in the lumen of a single airway, during inspiration or expiration. The air in the lumen is assumed to be incompressible and Newtonian. A steady state of the transports in the lumen is assumed (this steady state is obviously not the same depending on whether inspiration or expiration is considered). Indeed, both the residence time of the air in the generations contributing significantly to heat and water exchanges and the diffusion characteristic times in these airways are way smaller than the inspiration/expiration duration. This steady state is also a consequence of the fact that, as discussed below and shown in previous works (Karamaoun et al., 2018; Wu et al., 2014), the temperature profile in the wall of the airway can be considered at steady state during the entire respiratory cycle, due to the order of magnitude of several characteristic times. Finally, axisymmetry of the momentum, heat and mass transports is assumed.

r and z axes are defined in each airway (see figure S1). r (m) is the radial coordinate, with $r = 0$ at the center of the airway and $r = R$ at the Airway Surface Liquid (ASL)–lumen interface. z (m) is the axial coordinate along the airway. This z axis is oriented in the direction of the flow. $z = 0$ at one extremity of the airway and $z = L$ at the other one. Under a lubrication approximation as the flow in the airway is mainly directed in the z direction, the continuity equation as well as the momentum (along z and r), energy and mass conservation equations write:

$$\frac{1}{r}\frac{\partial}{\partial r}(rv_r) + \frac{\partial v_z}{\partial z} = 0 \tag{S3}$$

$$v_r\frac{\partial v_z}{\partial r} + v_z\frac{\partial v_z}{\partial z} = -\frac{1}{\rho}\frac{\partial p}{\partial z} + \frac{\nu}{r}\frac{\partial}{\partial r}\left(r\frac{\partial v_z}{\partial r}\right) \tag{S4}$$

$$\frac{\partial p}{\partial r} = 0 \tag{S5}$$

$$\frac{\partial}{\partial z}(v_z T) + \frac{1}{r}\frac{\partial}{\partial r}(rv_r T) = \alpha\frac{\partial^2 T}{\partial z^2} + \alpha\frac{1}{r}\frac{\partial}{\partial r}\left(r\frac{\partial T}{\partial r}\right) \tag{S6}$$

$$\frac{\partial}{\partial z}(v_z C) + \frac{1}{r}\frac{\partial}{\partial r}(rv_r C) = D\frac{\partial^2 C}{\partial z^2} + D\frac{1}{r}\frac{\partial}{\partial r}\left(r\frac{\partial C}{\partial r}\right) \tag{S7}$$

where $v_r(r,z)$ and $v_z(r,z)$ ($\mathrm{m\,s^{-1}}$) are the radial and axial components of the velocity at position (r,z) in the lumen. $p(r,z)$ ($\mathrm{kg\,m^{-1}\,s^{-2}}$), $T(r,z)$ (K) and $C(r,z)$ ($\mathrm{mol\,m^{-3}}$) are the pressure, the temperature and the water concentration at position (r,z) in the lumen, respectively. D ($\mathrm{m^2\,s^{-1}}$) is the diffusion coefficient of water in air and ρ ($\mathrm{kg\,m^{-3}}$), ν ($\mathrm{m^2\,s^{-1}}$) and α ($\mathrm{m^2\,s^{-1}}$) are the density, the kinematic viscosity and the thermal diffusivity of the air, respectively.

These transport equations are completed by boundary conditions. In the proximal generations of the lungs, the bifurcations in the bronchial tree create recirculations of the flow. This tends to homogenize the temperature and concentration fields in the lumen at the entrance of the airway. This also results in a non-established velocity profile at the entrance of the airway (i.e. differing significantly from a "Poiseuille" parabolic velocity profile). Moreover, in the proximal generations of the lungs, the flow-establishment length is usually significantly larger than the length of the airways. This causes a strong increase of the transfers in the airways (Pedley et al., 1970) and previous works have shown that considering a constant axial velocity on the entrance of the airway allows a good estimation of this enhancement of the transfers (Pedley et al., 1970; Wells et al., 2018). To take these elements into account, the following boundary conditions are applied at the inlet of the airway ($z = 0, \forall r \in]0, R[$):

$$T = T_{\text{in}} \text{ and } C = C_{\text{in}} \tag{S8}$$

$$v_z = v_{\text{av}} \text{ and } v_r = 0 \tag{S9}$$

T_{in} (K) and C_{in} ($\mathrm{mol\,m^{-3}}$) are the temperature and the water vapor concentration at the inlet of the airway, respectively. Both are assumed constant on this inlet. v_{av} ($\mathrm{m\,s^{-1}}$) is the flow rate of air in the airway divided by the area of its cross-section πR^2.

At the end of the airway ($z = L, \forall r \in]0, R[$), the axial diffusive fluxes of heat and water in the lumen are set to zero:

$$\frac{\partial T}{\partial z} = 0 \text{ and } \frac{\partial C}{\partial z} = 0 \tag{S10}$$

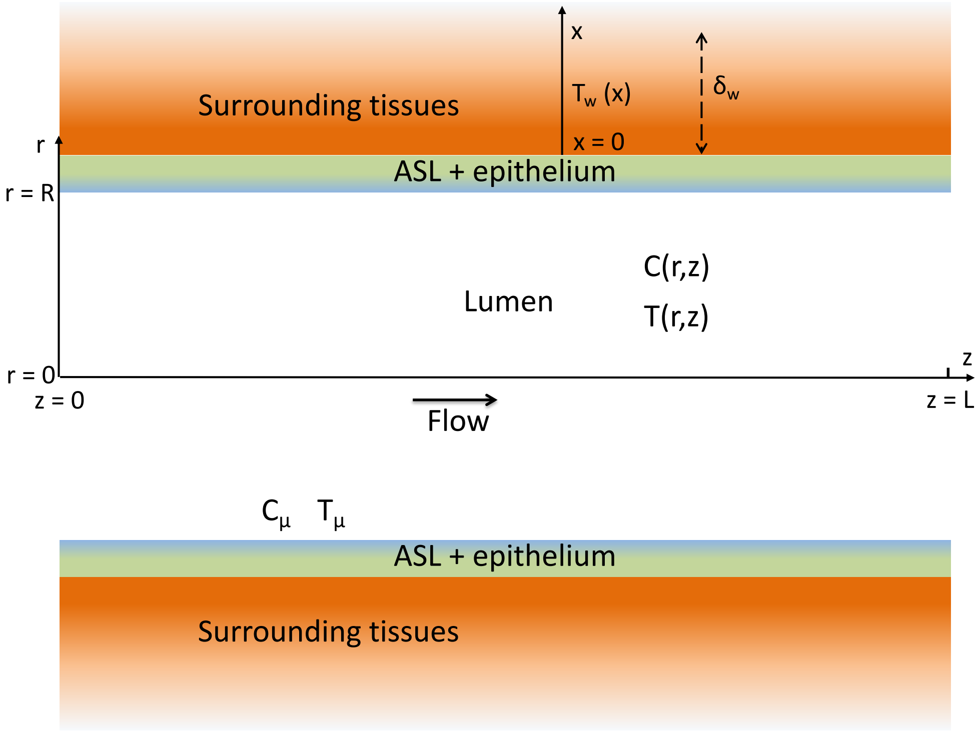

Figure S1. Schematic representation of an airway and the surrounding tissues. The figure is not on scale.

These boundary conditions are imposed because, for all the results presented in the core of the paper, the Péclet number of the flow in the airway, Pe = $v_{\mathrm{av}}L/D$, equal to the ratio of the characteristic time of axial transport by diffusion to the characteristic time of axial transport by convection, is well above 10 in almost all the airways of the bronchial tree. Actually, the smallest calculated value for this number is approximately 2 (in the case where the geometry described in the subsection "Adult in various situations" of the Results section is used, for breathing at rest and in the smallest airways of this geometry).

Moreover, at the end of the airway, the pressure p is arbitrary set to zero. This has no influence on the simulation results as it is the pressure gradient that appears in the momentum balance equations.

At the center of the airway ($\forall z \in]0, L[$, $r = 0$), the axisymmetry assumption gives:

$$v_r = 0\,,\ \frac{\partial v_z}{\partial r} = 0\,,\ \frac{\partial T}{\partial r} = 0 \text{ and } \frac{\partial C}{\partial r} = 0 \tag{S11}$$

Finally, at the ASL–lumen interface ($\forall z \in]0, L[$, $r = R$), a no-slip condition is used for the velocity:

$$v_r = v_z = 0 \tag{S12}$$

and it is assumed that a local equilibrium is achieved between the air and the ASL:

$$T = T_\mu \text{ and } C = C_\mu = C_{\mathrm{sat}}(T_\mu) \tag{S13}$$

where T_μ is the temperature of the ASL and C_μ is the concentration of water in the air in contact with the ASL–lumen interface. T_μ (and thus C_μ) is assumed to be independent of z. This is motivated by the

fact that, for all the results presented in the core of this paper, the dimensionless water concentration and temperature of the lumen show small variations (less than 0.1) in a single airway, during inspiration and expiration. Consequently, the energy extraction from the mucosa is limited in a single airway.

These equations can be averaged to give a macroscopic view of the heat and water transfers in the lumen of the airway. For this purpose, two averages can be defined on the flow cross-section of the lumen, a velocity average:

$$< y >= \frac{1}{\pi R^2 v_{\text{av}}} \int_0^R 2\pi y v_z r dr \tag{S14}$$

where y is either the temperature T or the water concentration C, and a space average:

$$< y >^* = \frac{1}{\pi R^2} \int_0^R 2\pi y r dr \tag{S15}$$

The velocity average of y on the lumen of the entire airway can also be introduced:

$$[y] = \frac{1}{\pi R^2 L v_{\text{av}}} \int_0^L \int_0^R 2\pi y v_z r dr dz \tag{S16}$$

Then, equation S7 is multiplied by $2\pi r$ and the resulting equation is integrated from 0 to R, yielding (with the second term vanishing due to the no slip and symmetry boundary conditions):

$$\frac{d}{dz}\left(\int_0^R 2\pi C v_z r dr\right) = 2\pi D R \left.\frac{\partial C}{\partial r}\right|_{r=R} + D\frac{d^2}{dz^2}\left(\int_0^R 2\pi C r dr\right) \tag{S17}$$

The diffusion flux of water vapor at the ASL–lumen interface can be classically rewritten by introducing a mass transfer coefficient k (m s^{-1}):

$$D \left.\frac{\partial C}{\partial r}\right|_{z,r=R} = k(C_\mu - < C >) \tag{S18}$$

Equation S17 can thus be rewritten as:

$$\pi R^2 v_{\text{av}} \frac{d < C >}{dz} = 2\pi R k(C_\mu - < C >) + \pi R^2 D \frac{d^2 < C >^*}{dz^2} \tag{S19}$$

If k is assumed independent of z, equation S19 can be integrated from $z = 0$ to $z = L$, yielding:

$$\pi R^2 v_{\text{av}} (C_{\text{out}} - C_{\text{in}}) = 2\pi R k L(C_\mu - [C]) - \pi R^2 D \left.\frac{d < C >^*}{dz}\right|_{z=0} \tag{S20}$$

with $C_{\text{out}} =< C >_{z=L}$.

Finally, equation S20 can be rearranged as:

$$\text{Sh} = \frac{Rk}{D} = \frac{1}{4}\frac{\text{Re Sc}}{\beta} \frac{C_{\text{out}} - C_{\text{in}} + \frac{D}{v_{\text{av}}} \left.\frac{d<C>^*}{dz}\right|_{z=0}}{C_\mu - [C]} \tag{S21}$$

with Sh, a Sherwood number, the dimensionless mass transfer coefficient between the ASL and the lumen, $\text{Re} = 2Rv_{\text{av}}/\nu$, the Reynolds number of the flow in the airway, $\beta = L/R$, the aspect ratio of the airway, and $\text{Sc} = \nu/D$, the Schmidt number of the air.

Now let us get back to equation S19. Results of numerical simulations of equations S3 to S13 (see below for more details) show that the second term of the right-hand side of this equation can be neglected, for all the situations considered in the core of this paper. If so, the resulting equation can be solved to give:

$$C_{\text{out}} = \frac{C_{\text{in}} - C_{\mu}}{\Psi} + C_{\mu} \tag{S22}$$

with

$$\Psi = \exp\left(4\beta \frac{\text{Sh}}{\text{Re Sc}}\right) \tag{S23}$$

a sole dimensionless number characterizing the ability of the transport phenomena within the lumen of the airway to condition the air. If $\Psi \to \infty$, $C_{\text{out}} \to C_{\mu}$ and, if $\Psi \to 1$, $C_{\text{out}} \to C_{\text{in}}$.

A similar reasoning for the heat transfer between the ASL and the lumen gives:

$$T_{\text{out}} = \frac{T_{\text{in}} - T_{\mu}}{\bar{\Psi}} + T_{\mu} \tag{S24}$$

with $T_{\text{out}} =< T >$ for $z = L$, and

$$\bar{\Psi} = \exp\left(4\beta \frac{\text{Nu}}{\text{Re Pr}}\right) \tag{S25}$$

with $\text{Pr} = \nu/\alpha$ the Prandtl number of the air and Nu, a Nusselt number, the dimensionless heat transfer coefficient, defined as $\text{Nu} = Ru/\lambda$, with λ ($\text{W m}^{-1}\,\text{K}^{-1}$) the thermal conductivity of the air and u ($\text{W m}^{-2}\,\text{K}^{-1}$) the heat transfer coefficient between the ASL and the lumen:

$$\lambda \left.\frac{\partial T}{\partial r}\right|_{z,r=R} = u(T_{\mu} - < T >) \tag{S26}$$

Similarly to the evaluation of Sh, Nu can be calculated as follows (if u is assumed constant):

$$\text{Nu} = \frac{1}{4}\frac{\text{Re Pr}}{\beta}\frac{T_{\text{out}} - T_{\text{in}} + \frac{\alpha}{v_{\text{av}}}\left.\frac{d<T>^*}{dz}\right|_{z=0}}{T_{\mu} - [T]} \tag{S27}$$

A dimensional analysis of the transport equations S3–S7 and their boundary conditions S8–S13 show that, at a fixed value of Sc (here equal to 0.63), Sh is a sole function of Re and $\beta = L/R$. Moreover, at a fixed value of Pr (here equal to 0.72), Nu is a sole function of Re and of β. Therefore, numerical simulations of equations S3–S13 (with for instance $C_{\text{in}} = T_{\text{in}} = 0$ and $C_{\mu} = T_{\mu} = 1$) can be used, with equations S21 and S27, to construct correlations relating Sh and Nu to Re and β: $\text{Sh} = f(\text{Re}, \beta)$ and $\text{Nu} = g(\text{Re}, \beta)$. These numerical simulations are performed with a discretization procedure written in Wolfram Mathematica 12. Second order upwind schemes are used for the convective terms, while the diffusion terms are discretized using a second order centered scheme. A first order scheme is used to discretize the pressure gradient. The mesh independency of the results has been checked. For this purpose, 200 discretization points are used in the z direction and 300 points are used in the r direction. Results of these simulations are presented in the

core of the paper (see figure 3), regarding the correlation Sh $= f(\mathrm{Re}, \beta)$. Notably, this figure shows that, when $\mathrm{Re}/\beta > 1$, Sh can be well approximated by Sh = $1.5 + 0.4\sqrt{\mathrm{Re\ Sc}/\beta}$. As Pr and Sc are close to each other and as the heat and water vapor transport equations (equations S6 and S7) are similar, there is a strong similarity between heat and mass transfers in the lumen of the airway and, basically, Nu and Sh are quite close to each other. Notably, when $\mathrm{Re}/\beta > 1$, Nu can be well approximated by Nu = $1.5 + 0.4\sqrt{\mathrm{Re\ Pr}/\beta}$.

Heat Transport in the Wall of the Airway

The equations developed in the previous section have to be completed by equations describing the transport phenomena in the bronchial wall and at the ASL–lumen interface (i.e. equations allowing evaluating C_μ and T_μ).

First of all, different phenomenological considerations must be introduced.

As sketched in figure S1, the wall of the airway is composed of the ASL (thickness of about 10 μm) lining a layer of epithelial cells (thickness of about 10 μm) and surrounded by tissues of varying nature. The heat transport in the wall of the airway is purely diffusive. Accordingly, an order of magnitude of the thickness of the wall of the airway impacted by heat transfer is $\delta_w = \sqrt{\alpha_w t^{\mathrm{insp}}}$, with α_w ($\mathrm{m}^2\ \mathrm{s}^{-1}$) the thermal diffusivity of the tissues composing the airway wall. The value of δ_w for a human adult at rest ($t^{\mathrm{insp}} \simeq 2$ s) is close to 500 μm. For all the results presented in the core of this paper, we have checked that $R \gg \sqrt{\alpha_w t^{\mathrm{insp}}}$ in the airways with a significant contribution to heat and water exchanges (and, thus, we have also $L \gg \sqrt{\alpha_w t^{\mathrm{insp}}}$). Moreover, as T_μ is assumed independent of the axial position in the airway, the same is assumed for T_w (K), the temperature in the bronchial wall. All these elements imply that the heat transport in the wall of the airway can be considered purely radial and described by a heat diffusion equation written in a 1D Cartesian axis system. For this purpose, x (m), an axial coordinate perpendicular to the airway wall, pointing outwards, with $x = 0$ at the lamina propria–epithelium interface, is introduced (see figure S1).

We assume that the tissues composing the wall of the airway are homogeneously vascularized by blood on a thickness larger than δ_w. Using the data presented by Montaudon et al. (2007), we have checked that, for all the results presented in the core of this paper for a human adult, this assumption is valid in the airways with a significant contribution to heat and mass transfer. We can introduce a characteristic time of the blood circulation in the tissues composing the wall of the airway, t_w (s), defined as the ratio of the volume of these tissues to the blood flow rate within them. For a human adult at rest, an order of magnitude of t_w can be evaluated. Indeed, at rest, the ratio of the volume of blood in the body to the cardiac flow rate is of the order of one minute. It can be assumed that t'_w (s), the ratio of the volume of blood in the tissues composing the wall of the airway to the blood flow rate within them is of the same order of magnitude. Moreover, in a tissue, the volume fraction of blood, ϕ, is typically 2–4$\%$ (Hindel et al., 2017). Consequently, at rest and for a human adult, the order of magnitude of $t_w = t'_w/\phi$ is between 1500 and 3000 s. When an effort is realized, t_w is decreased (by a factor 2–4), as the cardiac flow rate is increased. In the case of a vascularization defect, such as in asthma, t_w is increased. Our representation of the bronchial wall and our estimation of t_w are supported by several other works (Eisner and Martonen, 1989; McCullagh et al., 2010).

An important feature of the dynamics of heat transport in the wall of the airway can also be highlighted. First, as $t'_w \gg t^{\mathrm{insp}}$, the renewal of the blood in the wall of the airway takes place on a much larger time scale than the breathing time. Moreover, during inspiration, it can be easily evaluated that, per unit area of the ASL–lumen interface, the amount of heat that could be extracted by the ventilation from the wall

of the airway, whose order of magnitude is $t^{\text{insp}}k(C_{\text{sat}}(T_b) - C_0)\mathcal{L}_m$ (as mentioned in the introduction and demonstrated in the first section of this SM, the main cause of heat extraction from the mucosa is the energy used to evaporate water), with $\mathcal{L}_m$ (J mol^{-1}) the latent heat of vaporization of water, is way smaller than the amount of heat available in the wall, whose order of magnitude is $\sqrt{\alpha_w t^{\text{insp}}}\rho_w c_{p,w}(T_b - T_0)$, with ρ_w (kg m^{-3}) and $c_{p,w}$ (J kg^{-1} K^{-1}) the density and the heat capacity of the tissues composing the airway wall, respectively. Consequently, during a single respiratory cycle, neither the renewal of blood by the circulation nor the extraction of heat by ventilation have the ability to significantly modify the bronchial wall temperature. In other words, a steady temperature profile in the wall of the airway can be assumed over an entire respiratory cycle (this assumption is consistent with the numerical results presented by Wu et al. (2014)). It is controlled by the time average, over a whole respiratory cycle, of the temperature and water concentration in the lumen of the airway. It is worth to mention that, in a previous work (Karamaoun et al., 2018), we performed numerical simulations of the dynamics of the temperature of the tissues composing the wall of an airway, taking into account its time variation during inspiration and expiration. The results showed that, indeed, this temperature experiences small variations during a respiratory cycle.

Finally, we assume that there is no heat transfer limitation through the ASL and the epithelium, as they have a thickness way smaller than δ_w.

According to the different elements stated above, we propose the following equations to describe the heat transport in the wall of the airway:

$$\alpha_w \frac{d^2 T_w}{dx^2} + \frac{1}{t_w}(T_b - T_w) = 0 \tag{S28}$$

$$T_\mu = T_w(x = 0) \tag{S29}$$

$$\lim_{x \to \infty} T_w(x) = T_b \tag{S30}$$

$$\lambda_w \left.\frac{dT_w}{dx}\right|_{x=0} = \frac{1}{1+\gamma}\left(u^{\text{insp}}\left(T_\mu - [T]^{\text{insp}}\right) + \mathcal{L}_m k^{\text{insp}}\left(C_\mu - [C]^{\text{insp}}\right)\right) + \frac{\gamma}{1+\gamma}\left(u^{\text{exp}}\left(T_\mu - [T]^{\text{exp}}\right) + \mathcal{L}_m k^{\text{exp}}\left(C_\mu - [C]^{\text{exp}}\right)\right) \tag{S31}$$

with λ_w (W m^{-1} K^{-1}) the thermal conductivity of the tissues composing the wall of the airway. k^{insp} and k^{exp} are the mass transfer coefficients of the airway, during inspiration and expiration, respectively, while u^{insp} and u^{exp} are the heat transfer coefficients of the airway, during inspiration and expiration, respectively. The transfer coefficients might differ between the inspiration and the expiration, as the inspiration and expiration flow rates are different if $\gamma \neq 1$ (and, consequently, the Reynolds number of the flow in the airway is not the same at inspiration and expiration). $[C]^{\text{insp}}$ and $[T]^{\text{insp}}$ are the velocity averages of the water concentration and the temperature on the entire lumen of the considered airway during inspiration, respectively, while $[C]^{\text{exp}}$ and $[T]^{\text{exp}}$ are the velocity averages of the water concentration and the temperature on the entire lumen of the considered airway during expiration, respectively. $1/(1 + \gamma)$ is the ratio of the inspiration duration to the breathing cycle duration and $\gamma/(1 + \gamma)$ is the ratio of the expiration duration to the breathing cycle duration.

The two first terms of the right-hand side member of equation S31 characterize the heat withdrawal from the mucosa during inspiration to heat the air in the lumen and to evaporate water contained in the ASL. The two last terms characterize the exchanges between the lumen and the mucosa during expiration.

Introducing the solution of equation S28, with its boundary conditions S29 and S30, into equation S31 and rearranging gives, in a dimensionless form:

$$\Lambda(1-\tilde{T}_\mu) = \frac{1}{1+\gamma}\left(\tilde{C}_\mu - [\tilde{C}]^{\text{insp}}\right) + \frac{1}{1+\gamma}\Phi\left(\tilde{T}_\mu - [\tilde{T}]^{\text{insp}}\right) + \frac{\gamma}{1+\gamma}\frac{\text{Sh}^{\text{exp}}}{\text{Sh}^{\text{insp}}}\left(\tilde{C}_\mu - [\tilde{C}]^{\text{exp}}\right) + \frac{\gamma}{1+\gamma}\Phi\frac{\text{Nu}^{\text{exp}}}{\text{Nu}^{\text{insp}}}\left(\tilde{T}_\mu - [\tilde{T}]^{\text{exp}}\right) \quad \text{(S32)}$$

with $[\tilde{C}]^{\text{insp}}$, $[\tilde{T}]^{\text{insp}}$, $[\tilde{C}]^{\text{exp}}$, $[\tilde{T}]^{\text{exp}}$, $\tilde{C}_\mu$ and $\tilde{T}_\mu$ the dimensionless versions of $[C]^{\text{insp}}$, $[T]^{\text{insp}}$, $[C]^{\text{exp}}$, $[T]^{\text{exp}}$, C_μ and T_μ, respectively, Sh^{insp} and Sh^{exp} the values of the Sherwood number in the considered airway during inspiration and expiration, respectively, Nu^{insp} and Nu^{exp} the values of the Nusselt number in the considered airway during inspiration and expiration, respectively, and

$$\Lambda = \frac{\lambda_w (T_b - T_0)}{\mathcal{L}_m \frac{\text{Sh}^{\text{insp}} D}{R}(C_{\text{sat}}(T_b) - C_0)\sqrt{\alpha_w t_w}} \quad \text{(S33)}$$

$$\Phi = \frac{\text{Nu}^{\text{insp}}}{\text{Sh}^{\text{insp}}}\frac{\lambda}{D\mathcal{L}_m}\frac{T_b - T_0}{C_{\text{sat}}(T_b) - C_0} \quad \text{(S34)}$$

Finally, to complete the description of the dynamics at the level of the mucosa, another equation is written, the dimensionless version of equation S13, describing that the air in contact with the ASL is saturated with water:

$$\tilde{C}_\mu = \frac{C_{\text{sat}}(\tilde{T}_\mu(T_b - T_0) + T_0) - C_0}{C_{\text{sat}}(T_b) - C_0} \quad \text{(S35)}$$

4 COMPARISON BETWEEN THE COMPLETE MODEL AND THE SIMPLIFIED FRAMEWORK

Two examples of the comparison between the complete model and the simplified framework are given in figure S2. In this figure, the dimensionless concentration profiles in the lungs of an adult are plotted for a reference situation corresponding to case I in the core of the text (section “Adult in Various Situations”), i.e. a person at rest breathing in mild atmospheric conditions, and for a second case simulating an exercise. A very good comparison is observed between the approaches. Note that, to perform the comparison, the models are of course solved considering the same bronchial tree, more precisely using the morphometric values of L_i, R_i and n provided in the section “Adult in Various Situations” of the core of the paper. Hence it is not strictly speaking the entire simplified framework that is used but a simplified version of the complete model, obtained by neglecting the amount of energy extracted from the mucosa to heat the air (in front of the amount of energy extracted to evaporate water) and by linearizing the equation describing the liquid–gas thermodynamic equilibrium at the ASL–lumen interface.

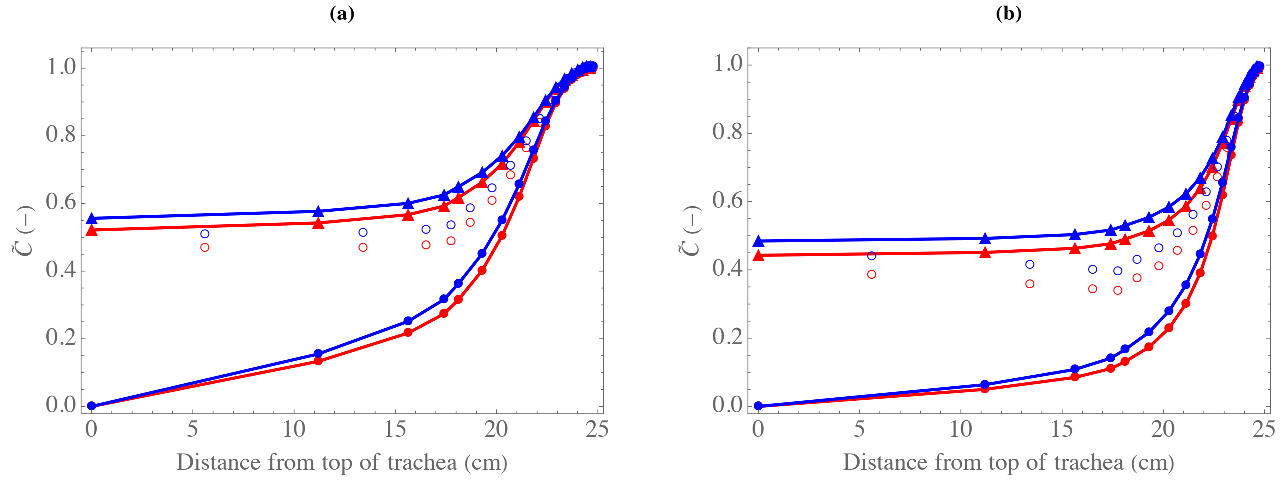

Figure S2. Dimensionless water concentration in the lumen, during inspiration (full circles) and expiration (triangles), as a function of the distance from the top of the trachea. Empty circles: values of $\tilde{C}_{\mu,i}$. Blue curves: complete model. Red curves: simplified framework. **(a)**: $Q^{\text{insp}} = 15\,\text{l}\,\text{min}^{-1}$, $t_w = 2000$ s, $\gamma = 1$, $T_0 = 33$°C and RH_0 = 0.9. **(b)**: $Q^{\text{insp}} = 60\,\text{l}\,\text{min}^{-1}$, $t_w = 1000$ s, $\gamma = 1$, $T_0 = 33$°C and RH_0 = 0.9.

REFERENCES

Eisner, A. D. and Martonen, T. B. (1989). Simulation of heat and mass transfer processes in a surrogate bronchial system developed for hygroscopic aerosol studies. *Aerosol Science and Technology* 11, 39–57

Hindel, S., Sohner, A., Maas, M., Sauerwein, W., Mollmann, D., Baba, H. A., et al. (2017). Validation of blood volume fraction quantification with 3d gradient echo dynamic contrast-enhanced magnetic resonance imaging in porcine skeletal muscle. *PLOS One* 12, –

Karamaoun, C., Sobac, B., Mauroy, B., Van Muylem, A., and Haut, B. (2018). New insights into the mechanisms controlling the bronchial mucus balance. *PLOS One* 13

McCullagh, A., Rosenthal, M., Wanner, A., Hurtado, A., Padley, S., and Bush, A. (2010). The bronchial circulation-worth a closer look: A review of the relationship between the bronchial vasculature and airway inflammation. *Pediatric Pulmonology* 45, 1–13

Montaudon, M., Desbarats, P., Berger, P., de Dietrich, G., Marthan, R., and Laurent, F. (2007). Assessment of bronchial wall thickness and lumen diameter in human adults using multi-detector computed tomography: comparison with theoretical models. *Journal of Anatomy* 211, 579–588

Pedley, T. J., Schroter, R. C., and Sudlow, M. F. (1970). The prediction of pressure drop and variation of resistance within the human bronchial airways. *Respiration Physiology* 9, 387–405

Sobac, B., Talbot, P., Haut, B., Rednikov, A., and Colinet, P. (2015). A comprehensive analysis of the evaporation of a liquid spherical drop. *Journal of Colloid and Interface Science* 438, 306–317

Warren, N. J., Crampin, E. J., and Tawhai, M. H. (2010). The role of airway epithelium in replenishment of evaporated airway surface liquid from the human conducting airways. *Annals of Biomedical Engineering* 38, 3535–3549

Wells, A. K., Jones, I. P., Hamill, I. S., and Bordas, R. (2018). The prediction of viscous losses and pressure drop in models of the human airways. *Respiratory Research* 34, –

Wu, D., Tawhai, M. H., Hoffman, E. A., and Lin, C. L. (2014). A numerical study of heat and water vapor transfer in MDCT-based human airway models. *Annals of Biomedical Engineering* 42, 2117–2131
